# Artificial Intelligence in Biomedical Sciences: A Scoping Review

**DOI:** 10.3389/bjbs.2025.14362

**Published:** 2025-08-05

**Authors:** Rasha Abu-El-Ruz, Ali Hasan, Dima Hijazi, Ovelia Masoud, Atiyeh M. Abdallah, Susu M. Zughaier, Maha Al-Asmakh

**Affiliations:** ^1^ Department of Biomedical Sciences, College of Health Sciences, QU Health, Qatar University, Doha, Qatar; ^2^ School of Arts and Sciences, Lebanese American University, Beirut, Lebanon; ^3^ Department of Biological and Environmental Sciences, College of Art and Science, Qatar University, Doha, Qatar; ^4^ College of Medicine, QU Health, Qatar University, Doha, Qatar; ^5^ Biomedical Research Center, Qatar University, Doha, Qatar

**Keywords:** biomedical sciences, artificial intelligence, scoping review, clinical, NAACLS

## Abstract

**Background:**

Artificial intelligence (AI) is increasingly playing important roles in healthcare diagnosis, treatment, monitoring, and prevention of diseases. Despite this widespread implementation of AI in biomedical sciences, it has yet to be characterized.

**Aim:**

The aim of this scoping review is to explore AI in biomedical sciences. Specific objectives are to synthesize six scopes addressing the characteristics of AI in biomedical sciences and to provide in-depth understanding of its relevance to education.

**Methods:**

This scoping review has been developed according to Arksey and O’Malley frameworks. PubMed, Embase, and Web of Science databases were searched using broad search terms without restrictions. Citations were imported into EndNote for screening and extraction. Data were categorized and synthesized to define six scopes discussing AI in biomedical sciences.

**Results:**

A total of 2,249 articles were retrieved for screening and extraction, and 192 articles were included in this review. Six scopes were synthesized from the extracted data: Scope (1): AI in biomedical sciences by decade, highlighting the increasing number of publications on AI in biomedical sciences. Scope (2): AI in biomedical sciences by region, showing that publications on AI in biomedical sciences mainly originate from high-income countries, particularly the USA. Scope (3): AI in biomedical sciences by model, identifying machine learning as the most frequently reported model. Scope (4): AI in biomedical sciences by discipline, with microbiology the discipline most commonly associated with AI in biomedical sciences. Scope (5): AI in biomedical sciences education, which was limited to only six studies, indicating a gap in research on the educational application of AI in biomedical sciences. Scope (6): Opportunities and limitations of AI in biomedical sciences, where major reported opportunities include efficiency, accuracy, universal applicability, and real-world application. Limitations include; model complexity, limited applicability, and algorithm robustness.

**Conclusion:**

AI has generally been under characterized in the biomedical sciences due to variability in AI models, disciplines, and perspectives of applicability.

## Introduction

Artificial Intelligence (AI) algorithms and models have been rapidly developed and trained to enhance various functions in biomedical operations. Biomedical sciences is one important healthcare profession that embraced AI in patient sampling, testing, interpretation, and diagnostic technologies [1, 2]. AI has been applied both virtually and physically to biomedical sciences, where virtual applications focus on information availability regarding testing and interpreting clinical results to support diagnosis and clinical decision-making, whereas the physical applications focus on robotics and tools that operate laboratory instruments with minimal human intervention [3–7].

Despite the wide application of AI in healthcare and biomedical sciences, its implementation and field translation are not always well characterized. The role of AI in biomedical sciences are being explored to further understand its implications [8]. For instance, Davenport 2019 reported that IBM developed the Watson AI model for precision medicine and cancer diagnosis, but its implementation was challenged by difficulties in training the model and its cost [9]. Abdulkareem 2021, discussed several obstacles to adopting AI in biomedical fields such as infection management, with major limitations including: data accessibility, need for large training data to develop the algorithms, difficulties in model updates, interdisciplinary expertise requirements, model validation, generalizability, high cost, and data privacy concerns [10, 11].

Despite these challenges, AI is increasingly being considered in clinical laboratory practice, including sample collection, processing, testing, and result interpretation. Many laboratories are now almost fully automated with minimal need for human input, and many of these automations imply AI working side-by-side with smart technologies [12]. In microbiology, AI has enabled the early and accurate detection of microbes, outbreak prediction, microbial growth monitoring, drug resistance profiling, and microbial genomics associated with disease development [13–15]. Similarly, AI-driven image analysis in haematology has helped to identify blood cell abnormalities, accelerating the diagnosis of diseases like leukemia [16]. Clinical chemistry laboratories also leverage AI to analyze plasma and amino acid profiles [17].

However, the application of AI in biomedical education, at both the pre-clinical and clinical levels, remains limited due to questions and concerns about its effectiveness, lack of standardization, and high costs [18]. Ethical concerns, including the risk of bias in AI models based on their training data, pose further challenges. Such biases may compromise educational outcomes if not carefully addressed [19–24]. Academic stakeholders also face challenges in making vital decisions regarding introducing AI in their curricula [25–28]. There were reported concerns of built-in biases and data confidentiality in AI models, this is contingent on the type of data used to train the model [23, 24].

This scoping review explores AI in biomedical sciences. Specific objectives are to synthesize six scopes addressing the characteristics of AI in biomedical sciences and to provide in-depth understanding of its relevance to education. The review answers two questions: what are the characteristics of AI in biomedical sciences? What are the applications of AI in biomedical sciences education?

The findings of this review will help in the evaluation of existing AI applications for potential use in pre-clinical and clinical education. A preliminary PubMed search revealed no similar reviews, indicating a lack of systematic evidence of AI applications in biomedical science. This study also offers foundational insights for academic stakeholders aiming to develop effective AI-enhanced strategies for biomedical sciences programs.

## Methodology

This scoping review was conducted according to Arksey and O’Malley frameworks [29]. We mapped the key concepts of AI research in biomedical sciences by summarizing and synthesizing the available sources of evidence. This study is a precursor of a forthcoming systematic review, in which we assessed the extent and nature of published research on this topic. We adhered to stages of scoping reviews outlined by Arksey and O’Malley 2005 [29], as well as the recommendations by Levac 2010 [30]. The stages included: developing two questions: what are the characteristics of AI in biomedical sciences? What are the applications of AI in biomedical sciences education? Followed by identifying the relevant studies by creating well-defined search strategies across three databases and evaluating their relevance. Then the selection criteria were carried out based on specific eligibility standards. The Qualitative data charting was performed using a detailed extraction sheet, following the recommendations of Ritchie and Spencer 2002 [31]. Finally, the data collection and synthesis included coding of keyword and thematic analysis, conducted in consultation with experts in Biomedical Sciences, resulting in the identification of six key scopes.

### Data Sources and Search Strategy

We conducted comprehensive searches of three databases: PubMed, Embase, and Web of Science. Our searches, completed on January 9, 2024, utilized broad search terms in titles and abstracts incorporating MeSH and Emtree terms expanded to include all subheadings, titles, and free-text terms (Supplementary Appendix S1). The citations obtained from our search strategy were imported to EndNote citation management software (v20.2.1). All team members contributed to the development of the search strategy, which was created in accordance with the Peer Review of Electronic Search Strategies (PRESS) 2015 checklist (Supplementary Appendix S2) [32].

### Study Selection and Eligibility Criteria

We included any publication of original research on the application of AI in the biomedical sciences. We excluded any publication not relying on primary data including, case reports, case series, editorials, expert opinion, commentaries, reviews, conference abstracts of peer-reviewed publications, and publications not relevant to educational, clinical, and research perspectives of biomedical sciences.

Disciplines within the biomedical sciences were defined according to the National Accrediting Agency for Clinical Laboratory Sciences (NAACLS) standards. Biomedical sciences referred to program studying the clinical disciplines of microbiology, chemistry, serology, haematology and blood banking, histotechnology, laboratory pathology, and laboratory management, all according to specific standards and policies for NAACLS accreditation [33–35]. NAACLS-accredited program graduates are eligible to take the American Society for Clinical Pathology (ASCP) board exam, which encompasses a broad spectrum of laboratory science topics essential for clinical competency [36–39].

### Data Extraction and Synthesis

Citations obtained following entry of our search strategy were imported into EndNote (v20.2.1) for screening. Duplicates were identified and deleted through EndNote library. The first screening entailed the identification of relevant or potentially relevant articles through title and abstract screening and was performed by two researchers. Then, the citations were rescreened, extracted, and double-extracted by three researchers and validated by a fourth reviewer. If a discrepancy was detected, consensus was reached with input from the corresponding author [40]. This scoping review was conducted systematically in accordance with the Preferred Reporting Items for Systematic Reviews and Meta-Analyses Extension for Scoping Reviews (PRISMA-ScR) checklist (Supplementary Appendix S3) [41].

Upon consensus between the research team, we adopted extraction variables relevant to our review. A spreadsheet for the extraction variables was developed according to the recommendation of Ritchie and Spencer 2002 [31], the included variables are: publication variables (author, citation, year of data collection, year of publication, country, and site type), study-related variables (study design, population type, sampling, gender, age group, mean age), and AI-related variables (name of AI, the purpose of AI, type of AI, description of AI aim, field, AI measure, effect size, reported effectiveness, advantages, limitations, conclusions, and real-life implementation).

The definition of AI in this review follows the World Health Organization’s guidance on the ethics and governance of AI for health, which is based on a recommendation by the Council on Artificial Intelligence of the Organization for Economic Co-operation and Development [42]. According to this definition, “*An AI system is a machine-based system that can, for a given set of human-defined objectives, make predictions, recommendations, or decisions influencing real or virtual environments. AI systems are designed to operate with varying levels of autonomy*” [42].

The protocol of this scoping review was registered in the Open Science Framework (OSF) on 4th June 2024 and was updated on 4th October 2024, with registration DOI: https://doi.org/10.17605/OSF.IO/4F2EZ.

### Data Synthesis

PRISMA flow chart was generated throughout the process of importing and extracting citations [43]. The extracted data were then thoroughly narratively mapped and summarized, followed by the construction of Six scopes. Scope (1): AI in biomedical sciences by decade. Scope (2): AI in biomedical sciences by region. Scope (3): AI in biomedical sciences by model. Scope (4): AI in biomedical sciences by discipline. Scope (5): AI in biomedical sciences education. Scope (6): Opportunities and limitations of AI in biomedical sciences. The latter scope involved coding keywords to generate categories of opportunities and limitations (Supplementary Appendix S6). The coding and categorizing of scopes were performed by three researchers, and uncertainties were discussed among all researchers until a consensus was achieved [2, 44]. Any unclear data from the citations was handled by discussion between the researchers and communication with the original authors whenever possible. Data were cleaned by four researchers and involved summarizing and reorganizing the data based on its relevance to this review.

## Results

### PRISMA Flow Chart

**FIGURE 1:**
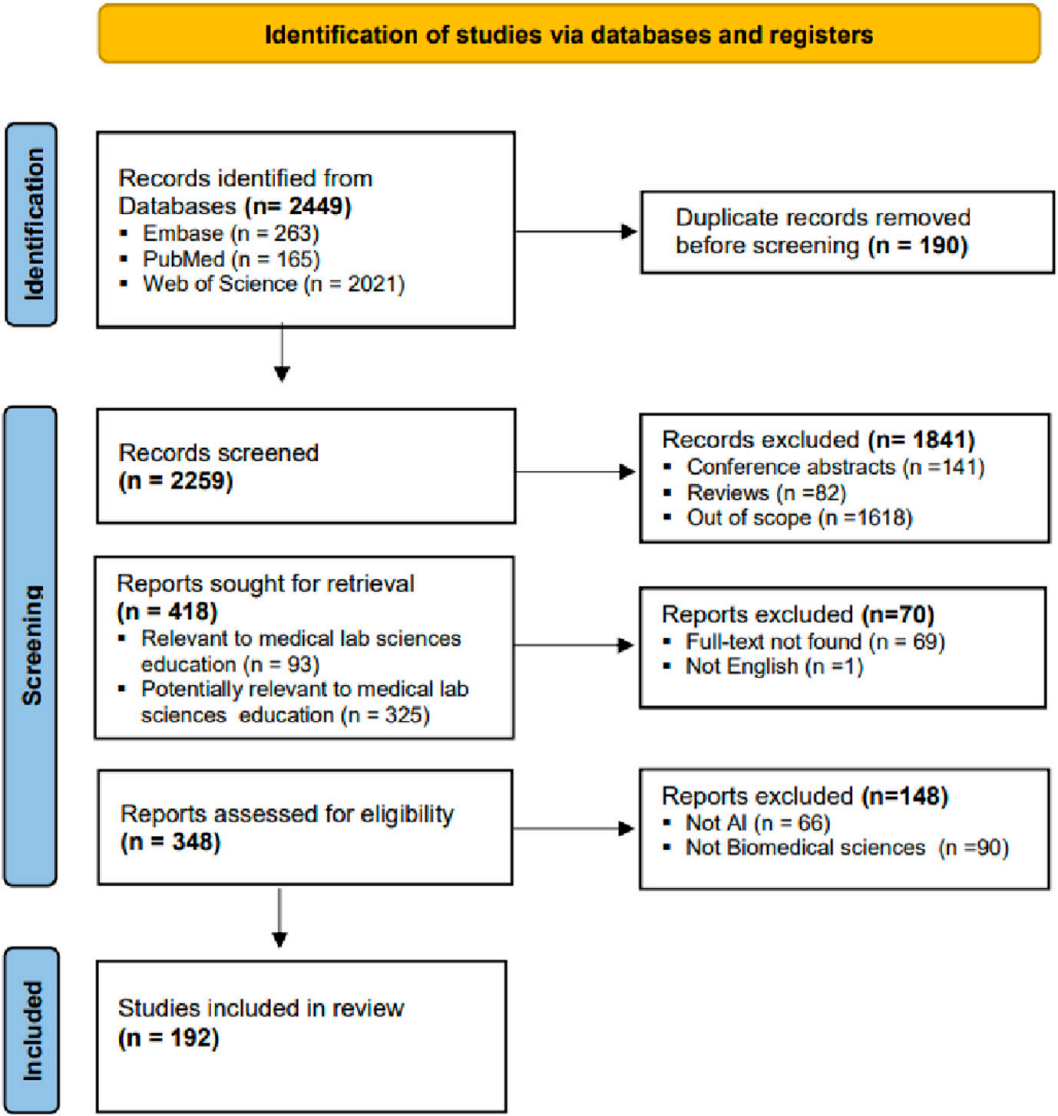
PRISMA flow chart.

We identified 2,449 articles, which were imported into our library: 263 from Embase, 165 from PubMed, and 2,021 from Web of Science. After removing 190 duplicates, a total of 2,259 articles remained for screening. The screening involved evaluating titles and abstracts, followed by full-text review, ultimately yielding 192 articles for data extraction. Articles were excluded for the following reasons: conference abstracts (141), reviews (82), out of scope (1618), full text not found (69), non-English language (1), not AI related (66), and not relevant to biomedical science (90) (Figure 1).

### Extraction Tables

The extracted key variables from the citations were organized by discipline (Supplementary Table S1). Discipline (1): Microbiology and infectious diseases. Discipline (2): Laboratory pathology, histotechnology, and cytogynecology. Discipline (3): Clinical chemistry. Discipline (4): Genetics and forensics. Discipline (5): Laboratory management. Discipline (6): Haematology and blood bank. Discipline (7): General laboratory sciences (*AI applications that are not limited to specific discipline and utilized across all laboratory sciences, as indicated in the extracted articles*). Discipline (8): Serology and immunology.

Additionally, a matrix was synthesized by mapping the first four scopes with the fifth scope “AI in biomedical sciences education,” in order to further identify the articles of educational perspective across the scopes (Supplementary Table S2).

### Qualitative Thematic Analyses

The following six scopes were synthesized from the key extraction variables. The narrative description provides an overview regarding AI in biomedical sciences. Scope (1): AI in biomedical sciences by decade; Scope (2): AI in biomedical sciences by region; Scope (3): AI in biomedical sciences by model; Scope (4): AI in biomedical sciences by discipline; Scope (5): AI in biomedical science education; Scope (6): Opportunities and limitations of AI in biomedical sciences.

#### Scope (1): AI in Biomedical Sciences by Decade

**FIGURE 2:**
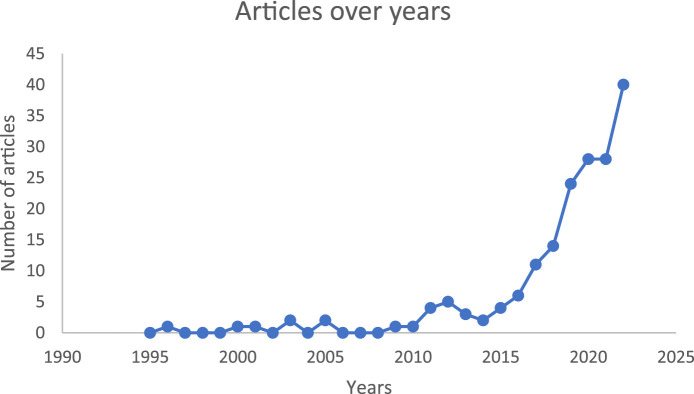
The number of articles published over time*. *Sharp increase in relevant publications over the last decade, highlighting increased interest in AI use in biomedical sciences.

The earliest article regarding use of AI in biomedical sciences was published in 1996 [45]. The extracted articles were classified by date into three major phases: pioneering phase (1996–2005), expansion phase (2006–2015), and prosperous phase (2016–2023) (Figure 2). In the pioneering phase, four articles attempted to explore AI in biomedical sciences, though none addressed the educational perspective. Two focused on clinical perspective (Liu 2001 and Prank 2005) [46, 47] while the other two examined the research perspective (Udelhoven 2000 and Giacomini 1996) [45, 48]. Liu 2001 aimed to develop a system capable of understanding and processing complex biomedical terms in written formats [46], while Prank 2005 predicted genotype from complex biochemical data, comparing linear and non-linear analytical methods to the performance of experienced clinicians [47]. Udelhoven 2000 established a hierarchical classification system for identifying bacteria using infrared spectra [48], and Giacomini 1996 employed AI to classify patients with HIV-1 using neural networks for improved clinical follow-up [45]. The main limitation of the models and algorithms during this decade was their complexity. Despite this, all studies reported high efficiency and accuracy with universal applicability [45]. All the studies in the pioneering phase were conducted in high-income countries including USA, Germany, Italy, followed by Taiwan and others.

In the expansion phase, 11 articles were published, two of them addressed the educational perspective (Munshi 2006 and Zheng 2015) [49, 50]. Munshi 2006 utilized simulation and visualization to focus on computational approaches for understanding molecular and cellular systems, reporting high reliability but limited applicability [49]. Zheng 2015 explored linking entities from unstructured full-texts of biomedical literature to 300 ontologies for automatic knowledge enrichment for scientific literature. Their approach had high accuracy and reliability, although the complexity of the algorithm was a limitation [50]. Five of the remaining articles discussed the research perspective, covering various aspects such as problem-solving, linguistics, handling narrative texts, and analytical schemes (Supplementary Tables S1, S2). The remaining four articles discussed the clinical perspective including clinical data assessment and diagnosis of infectious diseases (Supplementary Tables S1, S2). The majority of the opportunities reported in this decade highlighted high accuracy, reliability, and efficiency. However, the complexity of algorithms and limited model robustness were noted as significant limitations.

In the prosperous phase, 177 articles were published, a significant increase compared to the 11 published in the expansion phase and four in the pioneering phase. The majority, 122 articles, focused on the clinical perspective across various disciplines, with microbiology being the most discussed, followed by histotechnology, general laboratory sciences, haematology, and others (Supplementary Tables S1, S2). These articles primarily addressed the testing and diagnostic aspects of existing models. Fifty-one articles explored the research perspective, discussing the development and training of models to enhance research findings in biomedical sciences (Supplementary Tables S1, S2). Of all the articles in the prosperous phase, only four discussed the educational perspective Ambite 2019, Aparicio 2018, Henderson 2020, and Tota 2021 [51–54]. Ambite 2019 explored a tool that enabled computers to learn from data without programming, designing the ERuDIte framework to foster open-source educational resources on the web. Aparicio 2018 investigated how educators teaching biomedical subjects at the university level used intelligent information access systems such as BioAnnote, CLEiM, and MedCMap. Henderson 2020 explored the use of AI to improve the learning environment by supporting student engagement and activities. Tota 2021 proposed a telepresence robot kit with modules for teaching and conducting remote laboratory experiments.

Articles published during this phase highlighted enhanced efficiency, universal applicability, and real-world clinical relevance. However, algorithm complexity, limited model robustness, and moderate accuracy and reliability were reported as limitations.

#### Scope (2): AI in Biomedical Sciences by Region

One-hundred and thirty-four articles were from high-income countries (Supplementary Tables S1, S2), particularly USA (44 articles), followed by Germany (13 articles), Italy (10 articles), United Kingdome and Taiwan (9 articles each), and Japan (5 articles). Five of the AI models exploring the education perspective were conducted in high-income countries [49–53]. The majority of studies in these countries focused on the clinical perspective (84 articles), with a particular emphasis on microbiology and histotechnology. The opportunities reported in these studies included enhanced efficiency, universal applicability, and real-world clinical relevance. However, limitations such as algorithm complexity, limited model robustness, and moderate accuracy and reliability were also noted.

Fifty-four articles were from middle-income countries, notably China (25 articles), followed by India and Türkiye (7 articles each), Romania (3 articles), Thailand (2 articles), and others. Only one article from this group investigated AI from an educational perspective [54]. Forty articles explored AI from a clinical perspective and 13 articles from a research perspective. The majority explored AI in microbiology (23 articles) and general laboratory sciences (14 articles), with one in forensic medicine [55] and seven in haematology. Similar to studies from high-income countries, those from middle-income countries reported multiple benefits, including enhanced efficiency, universal applicability, and real-world clinical relevance. However, limitations such as algorithm complexity, limited model robustness, and moderate accuracy and reliability were also noted.

Only four articles were published from low-income countries: one article from each of Iraq [10], Bangladesh [56], Ethiopia [57], and Pakistan [58]. Three of the articles investigated AI from a research perspective, while one focused on a clinical perspective. No articles addressed the educational perspective. Three were in the field of microbiology and one in haematology. The articles reported opportunities such as enhanced efficiency, real-world clinical applicability, and high accuracy; however, the robustness of these AI models was limited (Supplementary Tables S1, S2).

#### Scope (3): AI in Biomedical Sciences by Model

Forty-seven articles explored AI in biomedical sciences using deep learning models. The majority (34 articles) examined AI from a clinical perspective, primarily in microbiology as well as laboratory pathology, while 13 articles focused on AI from a research perspective, mainly in general laboratory sciences. None of the deep learning models addressed the educational perspective.

In addition, 81 articles studied AI in biomedical sciences through machine learning models. Of these, 53 articles focused on clinical applications, primarily in microbiology, general laboratory sciences, and clinical chemistry, followed by histotechnology. Twenty-four articles investigated AI from a research perspective, with the general laboratory sciences being the dominant discipline, followed by microbiology and haematology. Three articles, Ambite 2019, Henderson 2020, and Tota 2021 examined the educational perspective around AI in laboratory sciences [51, 53, 54].

Twenty-five articles reported on hybrid AI applications in biomedical sciences, combining machine learning with deep learning, natural language processing, convolutional neural networks, or deep neural networks. The majority of this research focused on general laboratory applications, followed by microbiology, histotechnology, haematology, clinical chemistry, and forensic medicine. While most studies explored the clinical implications of hybrid AI models, none addressed their potential educational applications.

Natural language processing models were reported in seven articles, with six focusing on general laboratory disciplines and one on microbiology. Of these, four studied AI from a research perspective, one from a clinical perspective, and two from an educational perspective (Aparicio 2018 and Zheng 2015) [50, 52]. Neural networks were discussed in four articles, three from clinical perspective and one research, each addressing microbiology, chemistry, haematology, and serology.

**FIGURE 3:**
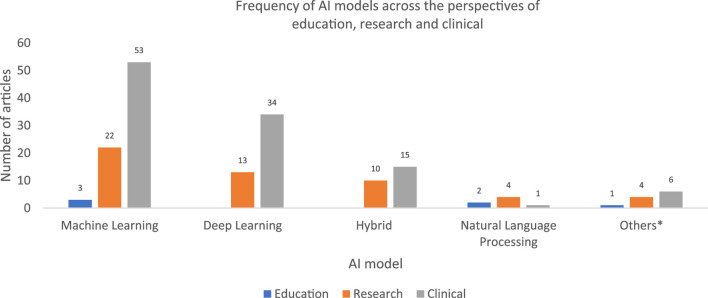
Bar chart showing the frequency of AI models in the reported articles*. *Others are automated AI-based diagnosis, generative adversarial networks, knowledge discovery and semantic analysis, and simulation and visualization.

Artificial neural networks and automated AI-based diagnosis method were each reported in two articles. Knowledge discovery, semantic analysis, and simulation and visualization were each discussed in one article, with the latter examining AI in biomedical sciences from an educational perspective (Munshi 2006) [49] (Figure 3).

#### Scope (4): AI in Biomedical Sciences by Discipline

Sixty-three articles focused on microbiology and infectious diseases. The main aims of these studies included pathogen identification, antimicrobial resistance prediction, automated image analysis and digital pathology, vaccine development, predictive modeling for disease outcomes and treatment, and biomedical text mining.

Twenty-four articles focused on laboratory pathology, histotechnology, and cytogynecology. The main aims of these studies included laboratory pathology and molecular analysis for cancer detection whether in body fluids samples or biopsies. Additionally, improving the diagnostic accuracy and consistency in cancer detection and therapy.

Ten articles focused on clinical chemistry, with the main aims including automation in urinalysis, biochemical and lipid analysis, clinical decision support systems, advanced applications in medical imaging, and automated scoring of laboratory assays.

Six articles focused on laboratory management, primarily addressing data management and clinical decision support systems.

Twenty articles examined haematology and blood bank applications, with key aims including automated blood smear analysis, abnormal blood cell identification, blood type identification, bone marrow cell classification, assessment of blood cell integrity in stored samples, screening and risk assessment for hematologic malignancies, blood clot detection, development of hematologic point-of-care testing systems, prediction of hemoglobin variants, and optimization of spectroscopic data analysis.

Fifty-eight articles explored general laboratory applications, those AI applications that are not limited to specific discipline and utilized across all laboratory sciences, as indicated in the extracted articles. The primary aims of those articles covered aspects including; conducting in-depth biomedical literature analyses, enhancing decision-making in intensive care unit laboratories, quality control applications, improving diagnostic accuracy through methods such as enhanced imaging, evaluating the effectiveness of AI use in biomedical laboratories, and proposing machine learning tools for non-expert biomedical technologists.

**FIGURE 4:**
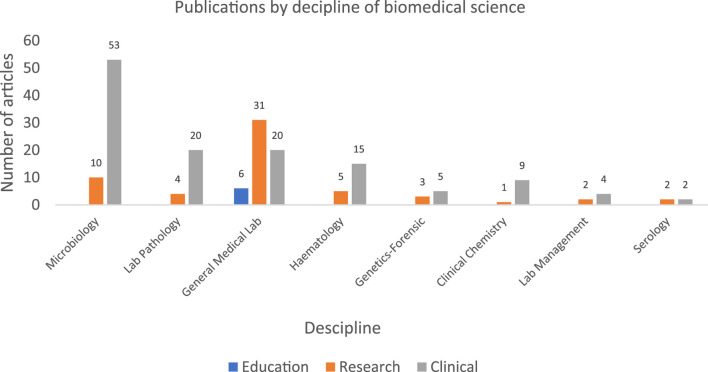
Bar chart showing the number of publications by discipline in biomedical sciences*. *Publications from clinical perspective dominated across all biomedical science disciplines, with microbiology, general medical laboratory and haematology leading overall output. Publications from educational perspective were minimal, highlighting a gap in academic-focused literature.

Four articles addressed serology, focusing on antigen identification and pathogen neutralization, improved immune cell treatment outcomes, classification of immune system diseases, and enhancing the diagnostic capabilities of serological tests through clinical cytometry (Figure 4).

#### Scope (5): AI in Biomedical Science Education: Further Depth

Of the extracted (192 articles), six reported the use of AI in biomedical sciences education [49–54].

The earliest paper investigating AI in education was a population-based, cross-sectional observational study by Munshi 2006. This study was conducted in the USA, employed simulation and visualization AI techniques within the general laboratory sciences discipline. The primary goal was to leverage computational and mathematical approaches to unravel the complexities of molecular and cellular systems. By simulating and visualizing biological processes at multiple scales, the study authors aimed to comprehend the complex biomedical processes, enable the observation and analysis of interactions at these scales, and facilitate hypothesis testing and model validation. While the study demonstrated high reliability, its applicability was limited [49].

A Romanian study by Tota 2021 proposed a computational algorithmic design for developing Telepresence Robotics, a technology to enable remote participation in courses and laboratories, allowing students to interact physically with teaching and laboratory equipment through robotic avatars. The robotic system incorporated AI to facilitate real-time measurements and error correction. The study advocated for a modular Telepresence Robot Kit, easy to assemble and adaptable to various educational settings. Machine learning AI was employed as a key tool in this innovative approach [54].

Another population-based study by Aparicio 2018, conducted in Spain using a qualitative mixed-method design, aimed to gather expert opinions on the use of intelligent systems for processing text to enhance learning activities in biomedical sciences. Interviews were conducted with educators using a questionnaire containing 66 predefined closed- and open-ended questions. The results revealed that teachers highly valued the integration of reliable information sources, bilingualism, and selective annotation of concepts [52]. This study focused on general biomedical laboratory disciplines and utilized a natural language processing AI tool.

A study by Zheng 2015, in the USA, explored a computational supervised entity-linking design. This unsupervised collective inference approach linked entities from unstructured full texts of biomedical literature to ontologies. The method leveraged the rich semantic information and structure in ontologies for similarity computation and entity ranking. Despite using no labeled data, the unsupervised approach outperformed a state-of-the-art supervised method that was trained on a large amount of manually labeled data. The study covered general biomedical laboratory disciplines and utilized a natural language processing AI tool. This method offered the potential to save scientists an enormous amount of time by providing easy access to specific data within a vast pool of information [50].

Another US-based study by Henderson 2020 employed a computational model-based approach to compare various machine learning-based affective models. The research aimed to recognize students' affective states within a game-based learning environment, utilizing competing feature-level and decision-level multimodal data fusion approaches. The results indicated that multimodal affect detectors, driven by posture- and interaction-based data, were effective in identifying emotional states. The AI tool examined in this study was a machine learning AI model [53].

A computational entity-linking study, conducted in the USA by Ambite in 2019, proposed ERuDITe, a platform that organized over 11,000 data science training resources, including courses, tutorials, and talks, specifically for biomedical researchers. Using machine learning, the platform tagged resources with relevant concepts from a newly created data science education ontology and linked people and organizations to public databases like DBpedia and ORCID. This approach provided access to personalized training and connected biomedical researchers to linked educational resources in data science [51].

#### Scope (6): Opportunities and Limitations of AI in Biomedical Sciences

Ambite in 2019 reported high accuracy and reliability for AI software like ERuDIte, with its comprehensive collection of over 11,000 data science training resources, including courses, video tutorials, and conference talks. The metadata for these resources was uniformly described using Schema.org, enhancing consistency and discoverability. However, the study discussed limitations related to algorithm complexity. Despite ERuDIte’s extensive data collection, the task of automatically identifying and organizing such diverse training resources is inherently complex, and the model’s robustness remains limited [51].

Aparicio 2018 highlighted the potential of AI in higher education, particularly in the biomedical field. Their research explored the benefits and challenges of integrating intelligent information access systems into active learning environments. They identified opportunities to enhance teaching practices, boost student engagement, and gain insights into educators' attitudes towards technological integration in biomedical education. While AI offers significant potential, the study also acknowledged limitations such as algorithm complexity, limited data sources, and technical constraints [52].

Tota 2021 proposed a telepresence robot kit containing easily assembled modules adapted for various teaching situations, specifically for the development of remote laboratory experiments. Users could create their own designs and adapt the electronic components and software to their needs through 3D printing and reprogrammable development boards. With components tailored to each situation, both simple laboratory tasks and complex operations, such as transporting and monitoring laboratory samples, could be performed remotely in real time, offering universal applicability. While this study advocated for telepresence in remote laboratory experiments, it acknowledged the complexity of the algorithm and the limited applicability of the AI software. The use of AI for text detection and recognition is challenged by the need for diverse requirements across different scenarios e.g., text in street scenes for robot navigation vs. receipts for optical character recognition (OCR) in financial departments. Also, difficulties in handling lower-quality or degraded data e.g., scanned legacy books in Google Books [54].

The approach presented by Zheng 2015 significantly outperformed state-of-the-art entity-linking methods, even without using labeled data. This would save scientists, particularly those focused on keeping informed about research developments, an enormous amount of time. All methods were highly accurate and efficient. However, the disambiguation algorithm assumed that phrases within the same sentence or paragraph were related, potentially undermining the entity linking (EL) performance [50].

Henderson 2020 demonstrated that combining different types of data creates a more accurate model for understanding and predicting the unique signals of emotions. By merging these data types, the model developed a comprehensive view of learners, leading to more accurate predictions than using just one data type. However, this study proposed an AI model that focused solely on specific datasets, neglecting some important affective states due to limited available observations. Additionally, the model was designed for specific learning environments, limiting its generalizability and robustness [53].

Simulation and visualization, while powerful tools for understanding complex biological processes, have limitations in real-time clinical practice. Implementing interdisciplinary programs that integrate simulation and visualization can also present challenges, such as group heterogeneity and time constraints. However, Munshi 2006 demonstrated that simulation and visualization can offer significant benefits, such as enhanced comprehension of complex biological processes, the ability to observe and analyze interactions at multiple scales, facilitation of hypothesis testing and model validation, and insights into the emergent properties of biological systems [49].

## Discussion

This scoping review synthesized six scopes addressing the characteristics of AI in biomedical sciences and provided in-depth understanding of its relevance to education. We summarized articles reporting on the implications of AI in biomedical sciences. Out of 2,249 publications, only 192 met our eligibility criteria and reported AI in one of the disciplines recognized by the NAACLS accreditation standards for biomedical sciences program and the disciplines of the ASCP board exam for biomedical scientists [34, 35, 37–39]. Of the 192 articles, only six directly addressed the educational perspective of their AI tools. The integration of AI in biomedical sciences dates back to 1996, when Giacomini proposed the use of a deep neural network for the clinical classification of HIV-1 patients based on P-24 and CD4 count [45].

There was a sharp increase in the number of articles published during the recent prosperous phase compared with earlier years. The AI tools reported in this phase covered all types of AI approach, including machine learning, deep learning, neural networks, and simulation and visualization, across various biomedical science disciplines. This trend aligns with Bohr 2020, who emphasized the need for healthcare institutions to keep pace with technological advances in healthcare and the growing demand for improved healthcare delivery outcomes [59]. Similarly, high-income countries are advancing rapidly in AI technology due to factors such as greater feasibility of utilization, budget availability, digital data availability, and infrastructure readiness, which are known limitations in low-income countries [60].

Microbiology has emerged as a leading discipline in the adoption of AI in biomedical sciences. Egli 2020 highlighted the significant shift toward digital microbiology, where advanced laboratory technologies powered by machine learning algorithms are revolutionizing the field. This trend is further fueled by the growth of microbiology big data. The increased utilization of AI in microbiology promises significant benefits, including improved testing, diagnosis, and treatment of infectious diseases, while also supporting related sectors such as antimicrobial stewardship, public health surveillance, quality control, and sepsis management [61].

There is limited evidence regarding the application of AI in biomedical sciences from an educational perspective, with only six studies discussing AI in education. Several limitations associated with incorporating AI into education were identified. Pedro 2019 reviewed the complexity of integrating AI into curricula, educational policies, public policies, equity considerations, risk assessment, sustainability, ethical concerns, and infrastructure needs [62]. Furthermore, Kuleto 2021 discussed the costs associated with AI in higher education, including structural costs, IT costs per student, and staffing costs [63]. Da Silva recently proposed autonomous experimentation (AE) systems, also known as self-driving laboratories or materials acceleration platforms, which are capable of running a large number of experiments autonomously. However, one challenge discussed was convincing stakeholders from emerging economies to invest in AE systems [64]. Many healthcare professionals, educators, and politicians have advocated making data available while maintaining anonymity to facilitate its integration into AI systems [65].

Machine learning was the most commonly reported AI approach used in the extracted articles, followed by deep learning. In machine learning models, AI can learn and adapt without manual input, using statistical models and algorithms to interpret the data [66]. Deep learning is a combination of machine learning and artificial neural networks. It is a self-learning software that layers algorithms into neural networks, allowing the system to teach itself [48]. Machine learning can be used for both data description and prediction. Based on the data, it can also provide models that serve as benchmarks for comparing human-led experiments. Artificial neural networks are important as prediction tools and can be used to adjust educational resources according to the individualized learning needs of students [67]. Deep learning-based algorithms can be combined with facial recognition software to assess students' attentiveness in online classes on platforms like Zoom, Google Classroom, and others [68]. Natural learning processing is an AI software model that enables computers to understand and communicate in human language, making interactions more intuitive and convenient [68]. Other AI models, such as convolutional neural networks, simulation, and visualization, can be used in a similar way.

The new era of AI in biomedical education should focus on incorporating AI systems into curricula and examining their application in real-life settings. Some initiatives have already begun, such as at Harvard Medical School, which has developed AI software capable of screening for a wide range of cancers and which can also be used for educational purposes [69]. Carnegie Mellon University is developing an AI-based voice pilot that will allow humans to communicate with robots [70], and the University of Southern California is using AI for teaching, learning, and campus research [71]. Hong Kong is incorporating AI education, AI ethics, and the social impact of AI into its school curriculum [72]. In India, the state of Andhra Pardesh is establishing two boards tasked with incorporating the use of AI in higher education [73]. The UK government has recognized the potential of generative AI tools like ChatGPT to support educators, initiating collaborations with educational stakeholders to explore how these tools can be used to analyze and structure information, ultimately aiding teachers in their work [74].

### Clinical and Research Studies With Potential Implications for Education

Alachram 2021 conducted a study at the University Medical Center, Taiwan, developing a Word2Vec approach using a corpus of 16 million PubMed abstracts. This approach helped to narrow down specific terms by leveraging this large database [75]. Anthony Q 2021 investigated deep learning models in clinical settings for super-resolution (DSLR), an emerging trend in response to the growing need for high-resolution images in machine learning/deep learning applications. The technique has shown promise for biomedical imaging, surveillance, and microscopy, with potential applications in education [76]. Deep learning-based virtual staining techniques, as discussed in Bai 2023, have enabled rapid, cost-effective, and chemical-free histopathology, offering a powerful alternative to traditional histological staining methods that have been in use for over a century [77]. Bonatti 2022 discussed extrusion-based bioprinting (EBB), an easy-to-use hardware system capable of printing a wide variety of materials. The paper advocated for the use of machine learning in the quality control of EBB, which could help publishers disseminate knowledge for educational purposes [78]. Gao 2019 demonstrated that the Edge2Vec model, in the research setting, could exploit machine learning and deep learning to provide powerful analytics and tools for graph analysis across three biomedical domain tasks: biomedical entity classification, compound-gene bioactivity prediction, and biomedical information retrieval [79]. Goncalves 2020 also explored deep learning AI based on convolutional layers and bidirectional gated recurrent units for automatic classification of abstract sentences into their main elements at a research center [80]. Hill 2016 conducted a study in a German University clinic on the use of machine learning to collect, process, analyze, and visualize data in real time, integrating cloud capabilities to ensure accessibility and scalability [81]. The approach supported a wide range of applications in both sports and biomedical disciplines, enhancing the usability and effectiveness of the sensor system. In a Chinese University research center, Li 2022 utilized domain-tuning pre-trained language models to explore methods for integrating imprecise knowledge into prompt-tuning verbalization techniques within the context of biomedical text to stimulate the rich knowledge distribution.

Ultra-high-speed imaging serves as a foundation for modern biomedical science. Liu 2022 conducted a study in a research setting and proposed all-fiber imaging at high speeds, achieved by transforming two-dimensional spatial information into one-dimensional temporal pulsed streams using high intermodal dispersion in multimode fibers. This technique could detect high-quality content-aware images and also images of various types from slightly reduced-quality training data. The imaging technique incorporated deep learning and improved imaging techniques could ultimately lead to better educational outcomes in biomedical science [82]. Luo 2022 used deep learning to train a set of transmissive diffractive surfaces that all-optically reconstructed images of arbitrary objects, even when completely covered by unknown, random phase diffusers [83]. The AI model faced challenges with adaptation, however if these challenges are addressed, it could usher in a new era of imaging systems capable of seeing through at the speed of light without the need for digital computation. This breakthrough would open up numerous new applications in biomedical imaging, astronomy, astrophysics, atmospheric sciences, security, robotics, and beyond. Similarly, Meade 2009 used machine learning in a research setting to optimize the entire analytical scheme and maximize the predictive capacity of spectroscopic data [84]. Nguyen 2022 assessed deep learning and machine learning to overcome the limitations of traditional design approaches in polymer science [85]. Rivenson 2018 provided proof-of-concept of a deep learning-based framework to enhance mobile phone-based microscopy by creating high-resolution, denoised, and color-corrected images through a convolutional neural network via a deep learning approach. This enhancement was achieved by training a deep convolutional neural network using smartphone microscope images and corresponding benchtop microscope images of various specimens [86]. Chiu 2017 used an AI model of limited maturity in a university set-up to create a system with a sparse annotation set to train and evaluate many phenotypes at once, known as “bulk learning” [87]. Liu 2001 used machine learning to develop a system that can understand and process complex medical terms [46].

More recently, Cadamuro 2023 explored ChatGPT’s ability and usefulness for interpreting laboratory test results (185). Similarly, Kumari 2023 studied ChatGPT-3.5, Google Bard, and Microsoft Bing in solving haematology-related cases, comparing their performance using a natural language processing AI tool [88]. Although ChatGPT is not an evidence-based tool, it has been increasingly used to search for information and synthesize knowledge. However, ChatGPT may be an extremely useful educational tool if used in legitimate and regulated ways.

A strength of this review lies in the large number of articles contributing to the synthesized scopes, the diversity of AI models used across biomedical fields, and the use of standardized procedures, structured validation, and the integration of multiple scopes to understand the characteristics of AI in biomedical sciences from various perspectives. However, the limitations of this review include the challenge of dealing with various study designs that cannot be critically appraised in the same manner. The majority of articles included are computational studies focusing on modeling, logarithmic, empirical, or factorial designs. There is, however, limited evidence from population-based studies, such as observational or experimental studies, which would provide more translationally meaningful outcomes. Other challenges include the complexity of the information extracted, requiring an interdisciplinary team, and the fact that we did not appraise the quality of the studies. Additionally, there may be contamination between some sub-categories of the scopes, such as disciplines, AI models, opportunities, and limitations.

## Conclusion

AI in biomedical sciences has been underexplored due to variability in AI models, disciplines, study designs, and perspectives of applicability. While the majority of AI applications in biomedical sciences are focused on clinical and research perspectives, studies exploring AI in biomedical education remain scarce. Furthermore, the quality of publications examining AI in biomedical sciences requires further standardization to ensure consistency and reliability across studies.
