# Supplementary material for: Artificial Intelligence in Biomedical Sciences: A Scoping Review
Source: Br J Biomed Sci. 2025 Aug 5;82:14362. doi: 10.3389/bjbs.2025.14362 (PMC12360964; doi:10.3389/bjbs.2025.14362)
Supplement: Supplementary file 1 [file DataSheet2.docx]

Table 1 Extracted articles of biomedical by discipline

| Citation | Country | Name  of AI | Purpose  of AI | Type  of AI | Focus of aim | Opportunities | Limitations |
| --- | --- | --- | --- | --- | --- | --- | --- |
| Discipline (1): Microbiology and infectious diseases | | | | | | | |
| Abdulkareem KH, 2022 (1) | Iraq | Convolutional Neural Networks | Clinical | Deep Learning | Developing a diagnostic system using AI to enhance the accuracy of early-stage COVID-19 detection. | High Accuracy and Reliability, Real-world Clinical Applicability, Enhanced Efficiency | Limited Model Robustness, Algorithm Complexity and Limitation, Limited Applicability in real-time clinical practice |
| Beadell B, 2023 (2) | USA | Convolutional Neural Networks | Clinical | Machine Learning | Predicting outcomes in *Staphylococcus aureus* bloodstream infections by integrating bacterial growth images with patient platelet count data for ML-based prognostic models. | Enhanced Efficiency, Real-world Clinical Applicability | Algorithm Complexity and Limitation |
| Huang L, 2018 (3) | China | Convolutional Neural Networks | Clinical | Convolutional Neural Networks | Automate bacterial colony classification using deep convolutional neural networks, streamlining the pre-screening process in clinical bacteriology, and enhancing efficiency in bacterial species identification. | Enhanced Efficiency, Universal Applicability | Limited Model Robustness |
| Normand AC, 2022 (4) | France | Deep Learning | Clinical | Convolutional Neural Networks | Investigate whether MALDI-TOF MS equipment can be used to detect specific fungal clones within a population of various isolates of the same species, specifically Aspergillus flavus. | High Accuracy and Reliability | Algorithm Complexity and Limitation |
| AlJame M, 2021 (5) | Kuwait | Deep Forest | Clinical | Machine Learning | Create machine learning for COVID-19 diagnosis. | High Accuracy and Reliability, Real-world Clinical Applicability, Enhanced Efficiency | Limited Model Robustness |
| Al-Mualemi BY, 2021 (6) | China | Convolutional Neural Networks | Clinical | Deep Learning | Implement a machine learning technique for predicting septic shock. | High Accuracy and Reliability, Real-world Clinical Applicability | Limited Model Robustness |
| Asakura K, 2018 (7) | Japan | MALDI-TOF | Clinical | Machine Learning | Identifying a type of antibiotic-resistant *Staphylococcus aureus.* | High Accuracy and Reliability, Real-world Clinical Applicability | Limited Model Robustness |
| Babenko D, 2021 (8) | Kazakhstan | Fast-and-Frugal Trees | Clinical | Machine Learning | Develop a method for efficiently distinguishing between bacterial and enteroviral meningitis in children. | Real-world Clinical Applicability, High Accuracy and Reliability | Limited Model Robustness |
| Benkwitz-Bedford S, 2021 (9) | UK | Machine Learning | Clinical | Machine Learning | Use computer predictions to understand how different types of *E. coli* bacteria grow when exposed to low levels of antibiotics. | High Accuracy and Reliability, Real-world Clinical Applicability | Limited Model Robustness |
| Brinati D, 2020 (10) | Turkey | Convolutional Neural Networks | Clinical | Machine Learning | Explore the potential of utilizing CNN architecture to process one-dimensional blood parameter data into two-dimensional images for COVID-19 detection. | High Accuracy and Reliability, Enhanced Efficiency, Real-world Clinical Applicability | Limited Model Robustness |
| Chadaga K, 2022 (11) | India | Machine Learning | Clinical | Machine Learning | Improve COVID-19 diagnosis accuracy by using blood tests and machine learning, and reviewing existing machine-learning applications for diagnosing COVID-19. | Enhanced Efficiency, Real-world Clinical Applicability | Limited Model Robustness |
| Choi BK, 2023 (12) | South Korea | Extreme Gradient Boosting, TabNet | Clinical, Research | Machine Learning & Deep Learning | Early determination of the etiology of meningitis and encephalitis in patients, as well as to identify important variables in the classification process. | High Accuracy and Reliability, Enhanced Efficiency | Limited Model Robustness |
| Cihan P, 2022 (13) | Turkey | Machine Learning | Clinical, Research | Machine Learning | Predicting SARS-CoV-2 epitopes. These predicted epitopes are intended to aid in the design of non-allergenic, non-toxic, and antigenic peptides for effective vaccine development against COVID-19 and other SARS family viruses. | High Accuracy and Reliability, Real-world Clinical Applicability, Enhanced Efficiency | Limited Model Robustness, Algorithm Complexity and Limitation |
| Court J, 2020 (14) | USA | Convolutional Neural Networks | Clinical | Convolutional Neural Networks | Improving mosquito-borne disease surveillance and management, particularly in identifying mosquito vectors such as those in the malaria-transmitting *Anopheles gambiae* complex. | High Accuracy and Reliability, Enhanced Efficiency | Limited Model Robustness |
| Çubukçu H, 2022 (15) | Turkey | Machine Learning | Clinical | Machine Learning | Enhance physicians' clinical judgment through accurate and efficient diagnostic assistance by developing a clinical decision support tool for diagnosing COVID-19 using machine learning models based on routine laboratory test results. | High Accuracy and Reliability, Real-world Clinical Applicability, Enhanced Efficiency | Moderate Model Accuracy and Reliability, Limited Model Robustness |
| Feng SY, 2022 (16) | China | PEARP and PEARR | Clinical | Deep Learning | Understand and predict the evolution of antibiotic resistance genes, focusing on blaCTX-M-14 in *Escherichia coli* strains, using a combination of experimental approaches and deep learning models. | High Accuracy and Reliability, Real-world Clinical Applicability | Limited Model Robustness |
| Ghamdi MAA, 2023 (17) | Saudi Arabia | VGG16, ResNet18 | Clinical | Deep Learning, Convolutional Neural Networks | Distinguishing COVID-19 patients from those with chest tuberculosis (TB) or pneumonia, as well as healthy individuals, based on symptoms and chest X-ray imaging. | High Accuracy and Reliability, Enhanced Efficiency | Algorithm Complexity and Limitation |
| Grossi M, 2019 (18) | Italy | Computer Vision | Research | Automated AI-based Diagnosis Method | Develop a computer vision sensor system to measure microbial concentration and estimate growth kinetics as an alternative to manual Plate Count Technique (PCT) analysis. | Enhanced Efficiency | Limited Applicability in real-time clinical practice, Limited Model Robustness |
| Hernandez B, 2017 (19) | UK | Decision Support System | Clinical | Machine Learning | Managing antimicrobial resistance (AMR) in clinical settings. It focuses on providing personalized diagnostics and therapeutic advice at the point-of-care (POC) and enhancing usability and knowledge transfer among clinicians. | High Accuracy and Reliability, Real-world Clinical Applicability | Algorithm Complexity and Limitation |
| Hou HX, 2021 (20) | China | Three Convolutional Neural Networks for classification: AlexNet, ZFNet, and VGG16 | Clinical | Convolutional Neural Networks | Automating the diagnosis of fungal keratitis using microscope images. | Enhanced Efficiency, Real-world Clinical Applicability | Algorithm Complexity and Limitation |
| Huang SW, 2020 (21) | Taiwan | Machine Learning | Clinical | Machine Learning | Develop prognostic models for severe dengue using ML, based on demographic information and clinical laboratory data of patients with dengue. | High Accuracy and Reliability, Enhanced Efficiency | Not Mentioned in Paper |
| Huang TS, 2023 (22) | Taiwan | Transfer Learning | Clinical | Deep Learning, Convolutional Neural Networks | Accurately identify filamentous fungi in medical laboratories using CNNs and transfer learning. | High Accuracy and Reliability, Real-world Clinical Applicability | Limited Model Robustness |
| Jansen P, 2022 (23) | Germany | Deep Learning | Clinical | Convolutional Neural Networks | Evaluate the effectiveness of a machine learning-based approach, specifically U-NET, in detecting fungal elements on digitized histologic sections of human nail specimens for diagnosing onychomycosis. | High Accuracy and Reliability | Limited Applicability in real-time clinical practice |
| Karadeniz I, 2015 (24) | Turkey | Rule-based AI | Research | Natural Language Processing | Automatically extracting bacteria habitat relations from biomedical text, thereby facilitating research in various areas such as health sciences, microbiology, and food processing. | High Accuracy and Reliability, Enhanced Efficiency | Algorithm Complexity and Limitation |
| Karunakaran V, 2022 (25) | India | Machine Learning | Clinical | Machine Learning | Analyze Raman spectral data from saliva samples to accurately differentiate between healthy individuals, COVID-19-infected patients, and COVID-19-recovered patients, and to classify different stages of patient recovery. | High Accuracy and Reliability, Universal Applicability | Algorithm Complexity and Limitation |
| Kong PH, 2022 (26) | Taiwan | Machine Learning | Clinical | Machine Learning | Early discrimination of *methicillin-resistant Staphylococcus aureus* (MRSA) strains from mass spectra in *Staphylococcus aureus* bacteremia (SAB) patients. | Enhanced Efficiency, Real-world Clinical Applicability | Limited Model Robustness |
| Kotepui M, 2015 (27) | Thailand | Machine Learning | Clinical | Machine Learning | Predicting malaria infection in suspected patients, aiming to enhance diagnosis and treatment. | High Accuracy and Reliability | Algorithm Complexity and Limitation, Limited Model Robustness |
| Kuo PC, 2020 (28) | Taiwan | Convolutional Neural Networks | Clinical | Convolutional Neural Networks | Assess the performance of an expert-level malaria detection algorithm, based on a CNN, using clinically validated image data sets (Taiwan Images for Malaria Eradication - TIME). | Real-world Clinical Applicability, High Accuracy and Reliability, Enhanced Efficiency | Limited Model Robustness, Algorithm Complexity and Limitation |
| Leite DMC, 2018 (29) | Switzerland | Machine Learning | Clinical | Machine Learning | Predicting interactions between phages and bacteria based on their genomes, offering a potential solution to antibiotic resistance challenges. | High Accuracy and Reliability, Enhanced Efficiency, Universal Applicability | Limited Applicability in real-time clinical practice |
| Mathison BA, 2020 (30) | USA | Convolutional Neural Network | Clinical | Convolutional Neural Networks | Augment the detection of intestinal protozoa in clinical parasitology laboratories by screening out negative trichrome slides while flagging potential parasites for manual confirmation. | High Accuracy and Reliability | Limited Applicability in real-time clinical practice |
| McGuire R, 2021 (31) | USA | Machine Learning | Clinical | Machine Learning | Develop a prediction model for carbapenem resistance | High Accuracy and Reliability | Limited Model Robustness |
| Murugan R, 2021 (32) | India | Deep Learning | Clinical | Deep Neural Networks | Develop an automatic diagnosis tool using DL to assist radiologists in quantitatively analyzing chest CT scan images for diagnosing lung infections in COVID-19 patients. | Enhanced Efficiency | Not Mentioned in Paper |
| Nakasi R, 2021 (33) | Uganda | Deep Learning | Clinical | Deep Learning | Automate the localization and count of *P. falciparum* parasites and White Blood Cells (WBCs) for effective malaria parasitemia determination. | High Accuracy and Reliability, Universal Applicability | Limited Model Robustness |
| Quoc VT, 2023 (34) | Vietnam | Random Forest | Clinical | Machine Learning | Evaluate the performance of ML models for predicting antibiotic resistance bacteria and resistance to antibiotics in intensive care unit (ICU) patients in Vietnamese hospitals. | High Accuracy and Reliability, Real-world Clinical Applicability | Limited Model Robustness |
| Ratzinger F, 2018 (35) | Austria | Random Forest | Clinical | Machine Learning | Establish predictive models using ML to assist clinicians in deciding when to conduct or avoid BC analysis based on the relevant risk of bacteremia. | High Accuracy and Reliability, Enhanced Efficiency | Limited Model Robustness, Algorithm Complexity and Limitation |
| Rohanian O, 2023 (36) | England | Machine Learning | Research | Machine Learning | Early detection of COVID-19 using ML, focusing on protecting sensitive patient information from adversarial attacks. | Enhanced Privacy Protection, High Accuracy and Reliability | Limited Model Robustness |
| Roland T, 2022 (37) | Austria | Machine Learning | Research | Machine Learning (no specific name) | Identify domain shifts in COVID-19 datasets, assess their effect on ML models, and emphasize the need for frequent re-training and reassessment. | High Accuracy and Reliability, Enhanced Efficiency | Limited Model Robustness, Moderate Model Accuracy and Reliability |
| Savardi M, 2018 (38) | Italy | Convolutional Neural Networks & Deep Learning | Clinical | Deep Learning | Automated detection of β-hemolysis in clinical microbiology images from Full Laboratory Automation systems. | High Accuracy and Reliability, Real-world Clinical Applicability | Limited Model Robustness |
| Shewajo FA, 2023 (39) | Ethiopia | Deep Learning | Clinical | Deep Learning | Enhance malaria parasite detection using a state-of-the-art (SOTA) object detection model. | Universal Applicability, Enhanced Efficiency, Real-world Clinical Applicability, High Accuracy and Reliability | Limited Model Robustness |
| Signoroni A, 2018 (40) | Italy | Convolutional Neural Networks & Deep Learning | Research | Convolutional Neural Networks | Assess the suitability and effectiveness of a DL approach for directly identifying pathogens on bacterial growing plates in the context of digital microbiology imaging. | High Accuracy and Reliability, Enhanced Efficiency | Algorithm Complexity and Limitation |
| Signoroni A, 2023 (41) | Italy | Deep Learning | Clinical | Convolutional Neural Networks | Develop a system for the global interpretation of diagnostic bacterial culture plates, including presumptive pathogen identification, in clinical microbiology. | High Accuracy and Reliability, Real-world Clinical Applicability | Algorithm Complexity and Limitation |
| Smith KP, 2017 (42) | USA | Machine Learning | Research | Deep Learning | Address the AST testing gap by developing a rapid and accurate method for determining antimicrobial susceptibility. | High Accuracy and Reliability | Algorithm Complexity and Limitation |
| Smith KP, 2018 (43) | USA | Convolutional neural network | Clinical | Convolutional Neural Networks | Develop and validate an automated classification methodology for blood-culture Gram stains. | High Accuracy and Reliability, Enhanced Efficiency | Algorithm Complexity and Limitation |
| Suratanee A, 2021 (44) | Thailand | Machine Learning | Clinical | Machine Learning | Predict human-*Plasmodium vivax* protein associations to identify potential drug targets for malaria treatment. | Universal Applicability, Enhanced Efficiency | Not Mentioned in Paper |
| Suri S, 2022 (45) | USA | Machine Learning | Clinical | Machine Learning | Develop a comprehensive ML method, IntegralVac, for predicting peptide binding affinity and immunogenicity in the context of vaccine design for COVID-19 and cancer. | High Accuracy and Reliability | Limited Model Robustness |
| Turra G, 2017 (46) | Italy | Deep Learning | Clinical | Deep Learning | Assess the suitability and effectiveness of a DL approach for directly identifying pathogens on bacterial growing plates using hyperspectral imaging. | Enhanced Efficiency | Algorithm Complexity and Limitation |
| Tyagin I, 2022 (47) | USA | Graph Mining and Transformer-Based Learning, | Research | Convolutional Neural Networks, Machine Learning | Develop automated hypothesis generation systems for COVID-19 research. | High Accuracy and Reliability, Real-world Clinical Applicability, Enhanced Efficiency | Algorithm Complexity and Limitation, Limited Model Robustness, Require Optimization |
| Udelhoven T, 2000 (48) | Germany | Artificial Neural Networks | Clinical | Artificial Neural Networks | Establish a hierarchical classification system using ANNs) for identifying bacteria based on Fourier transform infrared (FT-IR) spectra. | High Accuracy and Reliability | Algorithm Complexity and Limitation |
| Umer, 2020 (49) | Pakistan | Convolutional Neural Networks & Deep Learning | Research | Deep Learning | Enhance the automatic detection of malaria in blood smears by a novel stacked CNN. | High Accuracy and Reliability, Enhanced Efficiency | Limited Model Robustness |
| Wei ZY, 2023 (50) | China | Machine Learning | Clinical | Machine Learning | Identify distinct clinical signatures of fungal keratitis (FK) and develop a diagnostic model to differentiate FK from other types of infectious keratitis. | High Accuracy and Reliability, Real-world Clinical Applicability | Algorithm Complexity and Limitation |
| Wu LP, 2023 (51) | China | Support Vector Machine | Clinical | Machine Learning | Develop an AI diagnostic model with high sensitivity for early detection of bloodstream infections (BSI) in HIV-infected individuals. | High Accuracy and Reliability, Enhanced Efficiency, Real-world Clinical Applicability | Limited Applicability in real-time clinical practice, Limited Model Robustness, Algorithm Complexity and Limitation |
| Xu JB, 2023 (52) | UK | Machine Learning | Clinical | Machine Learning | Integrate AI and new diagnostic platforms into routine clinical microbiology laboratory procedures to achieve rapid and accurate identification of infectious fungi, thereby reducing turnaround time, and cost, and increasing diagnostic efficiency. | High Accuracy and Reliability, Enhanced Efficiency | Limited Model Robustness |
| Zeng Y, 2023 (53) | China | Machine learning | Clinical | Machine Learning | Evaluate and compare four MALDI-TOF-MS models for predicting the drug sensitivity of Klebsiella pneumoniae to imipenem. | Enhanced Efficiency, Universal Applicability, Real-world Clinical Applicability | Moderate Model Accuracy and Reliability |
| Zhang F, 2023 (54) | China | Machine learning | Clinical | Machine Learning | Predicting gram-positive and gram-negative bacteremia in patients based on routine laboratory parameters. | Real-world Clinical Applicability, Universal Applicability, Enhanced Efficiency | Limited Model Robustness |
| Zhang YM, 2023 (55) | Taiwan | Artificial Neural Networks | Clinical | Machine Learning | Develop an ANN model based on matrix-assisted laser desorption ionization time-of-flight mass spectrometry (MALDI-TOF MS) for the detection of *carbapenem-resistant Klebsiella pneumoniae* (CRKP) isolates. | Enhanced Efficiency, Real-world Clinical Applicability | Limited Model Robustness |
| Ceccon DM, 2023 (56) | Brazil | Machine learning | Clinical | Machine Learning | Develop a new methodology for rapid and precise label-free diagnosing of SARS-CoV-2 and COVID-19 infection in clinical samples, which combines.  Spectroscopic data acquisition and analysis via AI algorithms. | high accuracy and reliability, Enhanced Efficiency | Limited Model Robustness |
| Liu X, 2021 (57) | China | Support Vector Machine with Radial Basis Function kernel. | Clinical | Machine Learning | Classify and differentiate bacterial strains (MRSA and MSSA) based on mass spectrometry data. | Real-world Clinical Applicability | Moderate Model Accuracy and Reliability |
| Priyatharshini R, 2021 (58) | India | Convolutional Neural Networks | Clinical | Deep Learning | Differentiate COVID-19 from pneumonia records for effective and quick diagnosis of COVID-19 during the pandemic. | Real-world Clinical Applicability, High Accuracy and Reliability | Limited Model Robustness |
| Yasir M, 2022 (59) | Saudi Arabia | Machine Learning | Research | Machine Learning | Prediction of resistance to three commonly administered antibiotics for *P. aeruginosa* by training classifiers using quantitative GEXP information. | Real-world Clinical Applicability | Limited Model Robustness |
| Pettinati MJ, 2020 (60) | Canada | Machine Learning | Clinical | Convolutional Neural Networks | Evaluate the effectiveness of machine learning models, particularly XGBoost, in predicting sepsis. | Enhanced Efficiency, Real-world Clinical Applicability | Moderate Model Accuracy and Reliability |
| Richardson AM, 2013 (61) | Australia | Machine Learning | Clinical | Machine Learning | Investigate the effect of data pre-processing and ensemble-based classification methods on predicting hepatitis virus infection outcomes using routine pathology laboratory data. | High Accuracy and Reliability | Limited Model Robustness |
| Takayama T, 2011 (62) | Japan | Machine Learning | Clinical | Deep Learning | Predict the effectiveness of PEGIFN plus RBV therapy for individual patients with HCV genotype 1b | High Accuracy and Reliability | Algorithm Complexity and Limitation |
| Van DenBerg M, 2023 (63) | Netherlands | Machine Learning | Clinical | Machine Learning | Develop a predictive algorithm for early detection of late-onset sepsis (LOS) | High Accuracy and Reliability | Limited Applicability in real-time clinical practice |
| Ponce P, 2022 (64) | Mexico | Machine Learning | Research | Machine learning | Presents a low-cost robot, Robocov, designed as a rapid response against the COVID-19 Pandemic at Tecnologico de Monterrey, with implementations of artificial intelligence and the S4 concept for the design. | Enhanced Efficiency | Moderate Model Accuracy and Reliability |
| Nesaragi N, 2021 (65) | India | Machine Learning | Clinical | Machine Learning | Predict the onset of sepsis using electronic health record (EHR) data by applying tensor decomposition on correlation matrices of clinical covariates. | High Accuracy and Reliability | Algorithm Complexity and Limitation, Limited Model Robustness |
| Chiu PH, 2017 (66) | USA | Ensemble learning | Research | Machine Learning | Leveraging infectious diseases as a domain of study to tackle issues related to feature abstraction, scalability, and the establishment of accurate statistical models. | High Accuracy and Reliability, Enhanced Efficiency | Limited Model Robustness |
| Zurac S, 2022 (67) | Romania | Image-Augmentation Techniques | Clinical | Automated AI-based Diagnosis Method | Improve mycobacteria identification in Ziehl–Neelsen-stained slides for tuberculosis diagnosis using an AI-based method. | High Accuracy and Reliability | Limited Model Robustness, Limited Applicability in real-time clinical practice |
| Citation | **Country** | **Name**  **of AI** | **Purpose of AI** | **Type**  **of AI** | **Focus of aim** | **Opportunities** | **Limitations** |
| Discipline (2): Oncology, Histotechnology and Cytogynecoholgy | | | | | | | |
| Smith B, 2021 (68) | USA | Deep Learning | Clinical | Deep Learning | Explore the challenges and methods associated with incorporating DL algorithms into routine digital pathology workflow of biopsy slides. | High Accuracy and Reliability, Enhanced Efficiency | Algorithm Complexity and Limitation |
| Akbar S, 2019 (69) | Canada | Deep Convolutional Neural Networks | Clinical | Deep Learning | Explore automated methods for measuring tumor cellularity (TC) in breast cancer. | Real-world Clinical Applicability, High Accuracy and Reliability | Limited Model Robustness |
| Bai BJ, 2023 (70) | USA | Deep Neural Networks | Clinical | Deep Learning | Explore and explain recent advancements in virtual histological staining using deep learning techniques. | Enhanced Efficiency, High Accuracy and Reliability | Limited Model Robustness, Limited Applicability in real-time clinical practice |
| Brázdil T, 2022 (71) | Czech Republic | Machine Learning & Deep Learning | Clinical | Machine Learning & Deep Learning | Improve the identification and analysis of epithelial components within carcinomas by enabling large-scale guidance of AI methods to automatically detect epithelial regions in tumor samples. | High Accuracy and Reliability, Enhanced Efficiency, Real-world Clinical Applicability | Not Mentioned in Paper |
| Ceran Y, 2022 (72) | USA | Convolutional Neural Networks | Research | Deep Learning | Replace the time-intensive manual identification and counting of TNTs with a more efficient and accurate computational method. | High Accuracy and Reliability, Enhanced Efficiency | Moderate Model Accuracy and Reliability, Limited Model Robustness |
| Flinner N, 2022 (73) | Germany | Convolutional Neural Networks | Clinical | Convolutional Neural Networks | Developing an ensemble CNN using bagging to predict molecular subclasses directly from hematoxylin–eosin histology in gastric cancer (GC) patients. | High Accuracy and Reliability, Real-world Clinical Applicability, Enhanced Efficiency | Algorithm Complexity and Limitation |
| Ghahremani P, 2022 (74) | USA | DeepLIIF | Clinical | Deep Learning | Improve the qualitative or semi-quantitative reporting of biomarkers assessed by routine IHC staining of tissue in diagnostic pathology laboratories. | High Accuracy and Reliability | Algorithm Complexity and Limitation |
| Kundrotas M, 2023(75) | Lithuania | Machine Learning | Clinical | Machine Learning & Deep Learning | Enhance ML methods for detecting tumor-damaged tissues in histopathological images, improving early cancer diagnosis. | Enhanced Efficiency, Real-world Clinical Applicability | Algorithm Complexity and Limitation |
| Li RY, 2021 (76) | USA | Machine Learning | Clinical | Machine Learning | Classifying multi-omics human cancer data. | High Accuracy and Reliability, Real-world Clinical Applicability | Limited Model Robustness |
| Li XY, 2021 (77) | China | Subject-Predicate-Object triples | Research | Knowledge Discovery and Semantic Analysis | Propose a framework for Medical Know metrics using Semantic Predicate-Object (SPO) triples as knowledge units and uncertainty as the knowledge context, focusing on the field of medical knowledge extracted from scientific literature (validated by lung cancer dataset). | Enhanced Efficiency | Limited Model Robustness |
| Liang CW, 2021(78) | Taiwan | Convolutional Neural Networks | Clinical | Deep Learning, Convolutional Neural Networks | Predicting drug-sensitive mutations from images of gastrointestinal stromal tumors (GISTs), aiding in the treatment decision process. | High Accuracy and Reliability, Real-world Clinical Applicability | Limited Model Robustness |
| Naglah A, 2022 (79) | USA | Deep Learning | Clinical | Deep Learning | A cGAN-based deep learning model that transformed HE images into virtual MT images for accurate detection and quantification of fibrous tissue in liver specimens. | High Accuracy and Reliability, Enhanced Efficiency | Limited Model Robustness |
| Oskal KRJ, 2019 (80) | Norway | Convolutional Neural Network | Clinical | Convolutional Neural Networks | Develop an automated epidermis segmentation algorithm using CNNs as a crucial step in the creation of a Computer-Aided Diagnostic (CAD) system, to improve diagnostic efficiency, accuracy, and consistency. | High Accuracy and Reliability, Universal Applicability, Enhanced Efficiency | Limited Model Robustness, Limited Applicability in real-time clinical practice |
| Pellegrino E, 2021 (81) | France | Machine Learning | Clinical | Machine Learning | Develop a machine learning tool to classify NGS variants accurately and aid biologists in diagnosing cancer | High Accuracy and Reliability, Enhanced Efficiency | Algorithm Complexity and Limitation |
| Ruini C, 2021 (82) | Germany | Deep Learning | Clinical | Convolutional Neural Networks | Automated detection of squamous cell carcinoma (SCC) lesions in excised tissues using ex vivo confocal laser scanning microscopy (ex vivo CLSM) images. | High Accuracy and Reliability, Enhanced Efficiency, Real-world Clinical Applicability | Algorithm Complexity and Limitation |
| Saiz FS, 2021 (83) | USA | Machine Learning | Clinical | Convolutional Neural Networks, Machine Learning | Automate the analysis of clinical oncology literature and match articles to specific patient cohorts to answer questions about treatment efficacy, replicating a clinician's decision-making process. | High Accuracy and Reliability | Algorithm Complexity and Limitation |
| Simon O, 2018 (84) | USA | Support Vector Machine | Research | Machine Learning | Develop an automated method for localizing glomeruli in large histopathological whole-slide images (WSIs) of renal tissue sections and biopsies. | High Accuracy and Reliability, Enhanced Efficiency, Universal Applicability | Not Mentioned in Paper |
| Tellez D, 2018 (85) | Netherlands | Convolutional Neural Networks | Research | Convolutional Neural Networks | Develop an automated method to detect mitotic figures in breast cancer tissue sections. | Enhanced Efficiency | Limited Model Robustness |
| Tellez D, 2019 (86) | Netherlands | Convolutional Neural Networks | Clinical | Convolutional Neural Networks | Improve the generalization performance of CNNs trained on histopathology images by addressing the issue of stain variation across different laboratories. | Enhanced Efficiency, Real-world Clinical Applicability | Limited Model Robustness |
| Wu C, 2020 (87) | USA | Deep Learning and Support Vector Machine | Clinical | Machine Learning | Develop a ML method for efficiently distinguishing between genuine single-nucleotide variants (SNVs) and artifacts in nonformalin-fixed paraffin-embedded tumor specimens. | High Accuracy and Reliability, Universal Applicability | Limited Model Robustness, Moderate Model Accuracy and Reliability, Moderate Model Accuracy and Reliability |
| Zhang HR, 2020 (88) | UK | Deep Learning | Clinical | Deep Learning | Utilizing DL techniques to predict nuclear BAP1 (nBAP1) expression in uveal melanoma (UM) based on whole slide images (WSIs) of hematoxylin and eosin (H&E) stained sections. | Enhanced Efficiency, Universal Applicability, Real-world Clinical Applicability | Limited Model Robustness |
| Dhivya P, 2022 (89) | India | Deep Learning | Clinical | Deep Learning | Identifying brain tumors in brain MRI images using data augmentation, deep attention mechanism and hybrid firefly optimization techniques. | High Accuracy and Reliability, Enhanced Efficiency | Limited Model Robustness |
| Yacob F, 2023 (90) | Sweden | Convolutional Neural Networks | Clinical | Deep learning | Adopt the combination of graph neural networks and Transformers to detect and classify BCCs. | Real-world Clinical Applicability | Limited Model Robustness |
| You SX, 2019 (91) | USA | Deep Neural Networks | Clinical | Deep learning | Facilitate intraoperative assessment as well as pre- and post- operative diagnosis of biopsies and tissues for breast cancer. | High Accuracy and Reliability | Limited Model Robustness |
| Citation | **Country** | **Name**  **of AI** | **Purpose of AI** | **Type**  **of AI** | **Focus of aim** | **Opportunities** | **Limitations** |
| Discipline (3): Clinical chemistry | | | | | | | |
| Farrell CJL, 2022 (92) | Australia | Artificial Neural Network | Clinical | Deep Learning | Investigate how human supervision affects the performance of an artificial neural network (ANN) trained to identify wrong blood in tube (WBIT) errors using patient data from clinical chemistry applications. | High Accuracy and Reliability | Algorithm Complexity and Limitation |
| Gerl MJ, 2019 (93) | Finland | Machine Learning | Clinical | Machine Learning | To predict different measures of obesity based on plasma lipidome in a large population cohort using advanced machine learning modeling. | High Accuracy and Reliability, Enhanced Efficiency, Real-world Clinical Applicability | Algorithm Complexity and Limitation |
| Hwang S, 2021 (94) | Republic of Korea | Deep Learning | Clinical | Deep Learning & Natural Language Processing | To provide estimated LDL-C levels, using the DNN model to an electronic health record (EHR) system in real time (deep LDL-EHR) | Real-world Clinical Applicability | Moderate Model Accuracy and Reliability, Limited Model Robustness |
| Nagai T, 2022 (95) | Japan | Convolutional Neural Network | Clinical | Convolutional Neural Networks | Automate the classification of urinary sediment crystals, addressing the time-consuming and variable nature of manual classification performed under a microscope to achieve accurate and consistent results. | High Accuracy and Reliability, Real-world Clinical Applicability, Universal Applicability, Enhanced Efficiency | Limited Model Robustness |
| Streun GL, 2021 (96) | Switzerland | Machine Learning & Deep Learning | Clinical | Deep Neural Networks | Develop a ML model to detect urine sample manipulation, while simultaneously identifying prohibited drugs present in the sample. | High Accuracy and Reliability | Algorithm Complexity and Limitation |
| Yang ZY, 2019 (97) | China | Convolutional Neural Networks | Clinical | Convolutional Neural Networks | Automate the color classification of stool medical images using digital image processing and DL, specifically employing the StoolNet shallow CNN for accurate segmentation and classification. | Enhanced Efficiency, Universal Applicability | Limited Model Robustness |
| Wilkes EH, 2020 (98) | UK | Machine Learning | Clinical | Machine Learning | Automated Interpretation of Plasma Amino Acid Profiles. | Real-world Clinical Applicability | Moderate Model Accuracy and Reliability |
| Wilkes EH, 2018 (99) | UK | Machine Learning | Clinical | Machine Learning | Investigate ML-based CDS systems to support routine interpretation of biochemical profiling data within the clinical laboratory. | Real-world Clinical Applicability | Limited Model Robustness |
| Nguyen D, 2022 (100) | USA | Machine Learning | Research | Machine Learning & Deep Learning | Overcome the limitations of traditional design approaches in polymer science by integrating machine learning with coarse-grained molecular dynamics (CGMD) simulations. | High Accuracy and Reliability, Enhanced Efficiency | Limited Model Robustness |
| Erten M, 2023 (101) | **Australia** | **Swin-LBP model** | **Clinical** | **Machine Learning** | **Automate urine sediment analysis, crucial for diagnosing urinary tract and kidney diseases, overcoming time-consuming manual analysis with machine learning algorithms.** | **High Accuracy and Reliability, Enhanced Efficiency, Real-world Clinical Applicability** | **Limited Model Robustness** |
| Citation | **Country** | **Name**  **of AI** | **Purpose**  **of AI** | **Type**  **of AI** | **Focus of aim** | **Opportunities** | **Limitations** |
| Discipline (4): Genetics and forensic | | | | | | | |
| Birjan Z, 2023 (102) | Iran | ML: linear regression, support vector machine, kernels, decision tree, and extra-tree classifier, and deep learning models | Clinical | Machine Learning & Deep Learning | Investigate the association of Foxp3 single-nucleotide polymorphisms with systemic lupus erythematosus (SLE) and utilizes machine-learning and deep-learning methods to classify SLE patients from healthy individuals based on Foxp3 expression and associated genes. | Enhanced Efficiency, Universal Applicability | Limited Model Robustness |
| Buza K, 2016 (103) | Hungary | Semi-supervised learning | Research | Machine Learning | Develop a semi-supervised classifier that effectively handles gene expression data, even when labeled training samples are scarce or unavailable. | High Accuracy and Reliability, Enhanced Efficiency | Limited Model Robustness |
| Favalli V, 2021 (104) | Italy | Random Forest | Clinical | Machine Learning | Develop a ML based tool, RENOVO, capable of classifying variants as pathogenic or benign, providing a pathogenicity likelihood score (PLS) for enhanced interpretation. | High Accuracy and Reliability, Real-world Clinical Applicability | Limited Model Robustness |
| Manaka T, 2022 (105) | South Africa | Natural Language Processing | Clinical | Natural Language Processing & Machine Learning | To address the challenge of adapting NLP techniques in the health domain, specifically for the classification of causes of death (COD) using verbal autopsy (VA) reports. | High Accuracy and Reliability, Enhanced Efficiency | Limited Model Robustness |
| Marceddu G, 2019 (106) | Italy | Machine Learning | Clinical | Machine Learning | Distinguishing between Next Generation Sequencing (NGS) calls that require orthogonal confirmation and those that do not, aiming to decrease the workload needed for diagnosis. | Enhanced Efficiency | Limited Model Robustness |
| Prank K, 2005 (107) | Germany | Convolutional Neural Network and Support vector machines | Research | Machine Learning | Predict genotype from complex biochemical data using traditional linear analysis methods and novel non-linear analytical methods, comparing their performance to that of experienced clinicians. | High Accuracy and Reliability, Enhanced Efficiency | Limited Model Robustness |
| Price C, 2023 (108) | United States | Deep Support Vector Data Description (DeepSVDD) | Clinical | Deep learning | AMAnD is designed to identify and flag anomalous metagenomic samples that diverge from standard or “normal” signatures produced through routine metagenomic monitoring. | Enhanced Efficiency | Moderate Model Accuracy and Reliability |
| Wills JW, 2021 (109) | UK | DeepFlow neural network | Research | Deep learning | Show that imaging flow cytometry and deep learning image classification represents a capable platform for automated, inter-laboratory operation. | Enhanced Efficiency, Real-world Clinical Applicability | Moderate Model Accuracy and Reliability |
| Citation | **Country** | **Name**  **of AI** | **Purpose**  **of AI** | **Type**  **of AI** | **Focus of aim** | **Opportunities** | **Limitations** |
| Discipline (5): Laboratory management | | | | | | | |
| Flores E, 2023 (110) | Spain | Clinical Decision Support system | Clinical | Machine Learning | Demonstrate the adoption of a CDS system in the San Juan de Alicante Clinical Laboratory and to provide a protocol for implementation in other clinical laboratories. | High Accuracy and Reliability, Enhanced Efficiency | Not Mentioned in Paper |
| Gao Z, 2019 (111) | China | Machine Learning | Research | Machine Learning & Deep Learning | Validation of edge2vec model, which represents graphs considering edge, on three biomedical domain tasks: biomedical entity classification, compound-gene bioactivity prediction, and biomedical information retrieval. | High Accuracy and Reliability | Algorithm Complexity and Limitation |
| Gordon MM, 2012 (112) | Israel | Convolutional Neural Networks | Research | Machine Learning | Assess the impact of different data pre-processing and clustering algorithm choices on the formation of patient clusters and their reproducibility. | Enhanced Efficiency | Algorithm Complexity and Limitation |
| Sunil Mohan, 2018 (113) | USA | Delta Relevance Model | Research | Deep Learning | Measure the change in relevance of a document to a user's query over time to determine the relevance of search results to improve the accuracy and timeliness of search results. | High Accuracy and Reliability | Limited Model Robustness |
| Xue WY, 2020 (114) | China | Deep Learning | Clinical | Deep Learning | Deep-learning based approach that is used for textual information extraction from images of medical laboratory reports, which may help physicians solve the data-sharing problem. | Enhanced Efficiency | Algorithm Complexity and Limitation |
| Cope S, 2022 (115) | USA | Machine Learning | Clinical | Machine Learning | Provide workflow speedup (move, store and analyze vast amounts of data) on Intel® architecture through relevant health and life science using AI. | Enhanced Efficiency | Not Mentioned in Paper |
| Citation | **Country** | **Name**  **of AI** | **Purpose**  **of AI** | **Type**  **of AI** | **Focus of aim** | **Opportunities** | **Limitations** |
| Discipline (6): Hematology and blood bank | | | | | | | |
| Alam MM, 2019 (116) | Bangladesh | Machine Learning | Clinical | Deep Learning | Use a ML approach for automatic identification blood smear images | Enhanced Efficiency, High Accuracy and Reliability, Real-world Clinical Applicability | Limited Model Robustness |
| Barrera K, 2023 (117) | Spain | Convolutional Neural Networks | Clinical | Generative Adversarial Networks | Develop and validate SyntheticCellGAN (SCG), a system for generating artificial images of white blood cells, with a focus on realistic morphology to improve the training of diagnostic models for hematological diseases in clinical laboratories. | Real-world Clinical Applicability, High Accuracy and Reliability, Enhanced Efficiency | Limited Model Robustness |
| Barrera K, 2023 (118) | Spain | Automatic recognition models | Research | Deep Learning | Develop and evaluate a system to normalize color staining of blood cell images, making them consistent across different laboratories while preserving cell morphology. | Enhanced Efficiency | Limited Model Robustness |
| Eckardt JN, 2023 (119) | Germany | Machine Learning | Research | Machine Learning | Improve risk stratification and treatment allocation for AML patients and to compare their data-driven approach with traditional hypothesis-driven models to highlight the advantages of dynamic, data-centric methods. | Enhanced Efficiency | Limited Model Robustness |
| Hou J, 2023 (120) | China | Multi layer perception | Clinical | Deep Learning | Detect and identify clots and fibrins in serum images. | High Accuracy and Reliability, Real-world Clinical Applicability, Enhanced Efficiency | Algorithm Complexity and Limitation |
| Kumari A, 2023 (121) | India | Natural Language Processing | Research | Natural Language Processing & Machine Learning | Explore the capability of LLMs, namely, ChatGPT-3.5, Google Bard, and Microsoft Bing (Precise), in solving hematology-related cases and comparing their performance. | Enhanced Efficiency | Moderate Model Accuracy and Reliability |
| Lychagov VV, 2023 (122) | Russia | Machine learning | Clinical | Machine Learning | Explore the application of multiwavelength photoplethysmography (MW-PPG) in reflectance mode for noninvasive measurements of total hemoglobin concentration. | Enhanced Efficiency | Not Mentioned in Paper |
| Meade A, 2009 (123) | Ireland | Machine Learning | Research | Machine Learning | Optimize the complete analytical scheme and maximize the predictive capacity of the spectroscopic data. | High Accuracy and Reliability | Limited Model Robustness |
| Nozaka H, 2023 (124) | Japan | Deep Learning | Clinical | Deep Learning | Automatic identification of reactive lymphocytosis in blood smear images, with the ultimate goal of enhancing screening processes in clinical laboratories. | High Accuracy and Reliability, Real-world Clinical Applicability | Algorithm Complexity and Limitation |
| Peng KY, 2023 (125) | China | Convolutional Neural Network | Clinical | Convolutional Neural Networks | Enhance bone marrow cell classification precision with DAGDNet, integrating DAGs into DenseNet to improve identification of hematological disorders. | High Accuracy and Reliability, Real-world Clinical Applicability, Enhanced Efficiency | Algorithm Complexity and Limitation |
| Pfeil J, 2022 (126) | Germany | Deep Learning | Clinical | Deep Learning | Develop a point-of-care system for blood testing using a low-cost mobile microscope and smartphone-based algorithms. to detect and classify various blood cell types. | Real-world Clinical Applicability, Enhanced Efficiency, Universal Applicability | Limited Model Robustness |
| Rodellar J, 2022 (127) | Spain | Deep Learning | Clinical | Deep Learning | Automatically recognizing COVID-19 reactive lymphocytes (COVID-19 RL) in blood cell images, which serves as an indicator of better prognosis for COVID-19 patients. | High Accuracy and Reliability, Real-world Clinical Applicability, Universal Applicability | Limited Model Robustness |
| Rosales MA, 2022 (128) | Philippines | Machine Learning | Clinical | Machine Learning | Identifying the blood type of an individual using image processing and machine learning algorithms to minimize human error in manual blood typing. | High Accuracy and Reliability | Limited Model Robustness |
| Sadafi A, 2021 (129) | Germany | Convolutional Neural Network | Clinical | Convolutional Neural Networks | Predict the severity of sickle cell disease directly from Percoll gradient images. | High Accuracy and Reliability | Limited Model Robustness |
| Salama ME, 2022 (130) | USA | Deep Neural Networks | Clinical | Deep Neural Networks | Improve the efficiency of clinical laboratories in detecting minimal residual disease (MRD) in chronic lymphocytic leukemia (CLL) using deep neural networks (DNN). | High Accuracy and Reliability, Enhanced Efficiency, Universal Applicability | Algorithm Complexity and Limitation |
| Syed-Abdul S, 2020 (131) | South Korea | Machine Learning | Clinical | Deep Learning, Deep Neural Networks | Screen hematologic malignancies using Cell Population Data (CPD) and ML algorithms. | High Accuracy and Reliability | Limited Model Robustness |
| Uçucu S, 2022 (132) | Turkey | Artificial Neural Networks | Clinical | Machine Learning & Deep Learning | Predict hemoglobin variants, specifically identifying suspicious cases with HbS or HbD Los Angeles carriers state, using ML models | High Accuracy and Reliability, Real-world Clinical Applicability | Algorithm Complexity and Limitation |
| Zhou R, 2022 (133) | China | Deep Learning | Clinical | Machine Learning | Develop a highly accurate Delta Check (DC) for the detection of sample mix-ups in routine hematology tests. | Enhanced Efficiency, High Accuracy and Reliability, Universal Applicability | Limited Model Robustness, Limited Applicability in real-time clinical practice |
| A Branksy, 2021 (134) | Israel | Viscoelastic Focusing | Clinical | Machine Learning | Testing advanced hematology approach that is hemoscreen analyzer in different clinical setting and sample characteristics | High Accuracy and reliability, Enhanced Efficiency | Limited model robustness |
| Doan M, 2020 (135) | USA | Deep Learning | Clinical | Deep Learning | Identifying RBCs membrane integrity /degradation level in blood storage samples using deep learning and label free imaging flow cytometry. | Hight Accuracy and Reliability | Limited Model Robustness |
| Haung Z, 2020 (136) | USA | Machine Learning | Research | Machine Learning | Provide a highly sensitive, rapid and label-free method for timely diagnosis of heparin-induced thrombocytopenia (HIT). | Enhanced Accuracy and Reliability | Limited Model Robustness |
| Citation | **Country** | **Name**  **of AI** | **Purpose of AI** | **Type**  **of AI** | **Focus of aim** | **Opportunities** | **Limitations** |
| Discipline (7): General Laboratory sciences | | | | | | | |
| Islam MM, 2021 (137) | Taiwan | Deep Learning | Clinical | Deep Learning | Develop an automated tool for recommending lab tests. | Universal Applicability, Enhanced Efficiency | Limited Model Robustness, Algorithm Complexity and Limitation |
| Lippincott T, 2011 (138) | England | Natural Language Processing | Research | Natural Language Processing & Machine Learning | Identify and investigate the phenomenon of linguistic subdomain variation within the biomedical domain. | Enhanced Efficiency | Algorithm Complexity and Limitation |
| Alachram H, 2021 (139) | Taiwan | Convolutional Neural Networks | Research | Deep Learning & Natural Language Processing | Leverage the word2vec approach for generating word vector representations based on a large corpus of biomedical literature, particularly PubMed abstracts. | Enhanced Efficiency, Real-world Clinical Applicability | Limited Model Robustness |
| Ambite JL, 2019 (140) | USA | Natural Language Processing | Education | Machine Learning | Enable computers to learn from data without being programmed | High Accuracy and Reliability | Algorithm Complexity and Limitation |
| Anthony Q, 2021 (141) | USA | Machine Learning | Clinical | Deep Learning | Explore challenges associated with training single-image super-resolution (SISR) models | Enhanced Efficiency | Algorithm Complexity and Limitation |
| Aparicio F, 2018 (142) | Spain | Natural Language Processing & Machine Learning | Education | Natural Language Processing | Investigating how educators teaching biomedical subjects at the university level using AI | High Accuracy and Reliability | Limited Applicability in real-time clinical practice |
| Ayad A, 2022 (143) | Germany | Machine Learning and Deep Neural Networks | Clinical | Deep Learning | Predict near-future abnormalities in ICU lab values to assist clinical decision-making. | Enhanced Efficiency | Algorithm Complexity and Limitation |
| Barton S, 2021 (144) | Ireland | Convolutional Neural Networks | Clinical | Convolutional Neural Networks | Make Raman spectroscopy better at identifying diseases by improving how it deals with weak signals and noise. | High Accuracy and Reliability, Enhanced Efficiency | Limited Model Robustness |
| Bonatti AF, 2022 (145) | Italy | Deep learning-based control loop | Research | Machine Learning & Deep Learning | Implement a robust deep learning-based quality control loop for extrusion-based bioprinting. | High Accuracy and Reliability, Enhanced Efficiency, Universal Applicability | Algorithm Complexity and Limitation |
| Cadamuro J, 2023 (146) | Italy | Natural Language Processing | Research | Natural Language Processing | To test ChatGPT ability to interpret laboratory test results | Universal Applicability | Limited Applicability in real-time clinical practice, Moderate Model Accuracy and Reliability and Limited Model Robustness |
| Choi H, 2023 (147) | USA | Machine Learning | Research | Machine Learning | Allows researchers without machine learning or coding expertise to run supervised machine learning analysis through a clean web interface. It helps users to easily upload datasets, select models to train, and explore their data through intuitive visualizations. | Enhanced Efficiency | Algorithm Complexity and Limitation |
| Demirci F, 2016 (148) | Turkey | Convolutional Neural Networks | Clinical | Machine Learning, Convolutional Neural Networks | Develop an experimental decision algorithm model capable of efficiently and rapidly evaluating the results of biochemical tests with critical values by considering multiple factors concurrently. | High Accuracy and Reliability, Enhanced Efficiency | Algorithm Complexity and Limitation |
| Erdengasileng A, 2022(149) | USA | Natural Language Processing | Research | Natural Language Processing | Evaluate the effectiveness of information extraction methods in biomedical domain and facilitate their development as a community-wide effort. | Enhanced Efficiency | Moderate Model Accuracy and Reliability |
| Fernandez-Blanco E, 2015 (150) | Spain | Artificial Neural Networks | Research | Artificial Neural Networks | Automate ANNs development for biomedical data using EC (GAs, GP) to enhance efficiency and problem-solving capabilities. | High Accuracy and Reliability | Algorithm Complexity and Limitation |
| Fillmore N, 2019 (151) | USA | Machine Learning | Clinical | Machine Learning | Develop an interactive ML tool facilitating precise retrieval of specific lab data from electronic health records (EHRs) stored in large data warehouses. | High Accuracy and Reliability | Limited Model Robustness |
| Goncalves S, 2020 (152) | Portugal | Deep Neural Networks | Research | Deep Learning | Development and application of a deep learning classifier, based on a convolutional layer and a bidirectional gated recurrent unit, specifically tailored for classifying sentences within abstracts from the fields of biomedical science and computer science. | High Accuracy and Reliability | Limited Applicability in real-time clinical practice |
| He XY, 2021 (153) | China | hybrid neural network and MH-attention (Multi-Head attention) | Research | Deep Learning | Extract vocabulary-level features and Bidirectional Gated Recurrent Unit (BiGRU) to obtain contextual semantic information and properly extract the words describing the event types. | Enhanced Efficiency | Moderate Model Accuracy and Reliability |
| Hill M, 2016 (154) | Germany | Machine Learning | Clinical | Machine Learning | Provide a versatile, modular, and intelligent sensor platform capable of recording and analyzing physical parameters in real-time. | High Accuracy and Reliability | Algorithm Complexity and Limitation |
| Hong LX, 2020 (155) | China | Machine Learning | Research | Machine Learning | Introduce a new ML framework specifically for automatic extraction of biomedical relations from extensive repositories of literature to make predictions after considering all relevant statements. | Enhanced Efficiency | Not Mentioned in Paper |
| Hotz CS, 2005 (156) | USA | Machine Learning Algorithms | Research | Machine Learning | Compare the diagnostic accuracy of a rule-based expert system with two machine learning methods for categorizing body cavity effusions in companion animals. | Enhanced Efficiency | Algorithm Complexity and Limitation |
| Islam MM, 2020 (157) | Taiwan | Machine Learning & Deep Learning | Clinical | Deep Learning | Provide laboratory tests recommendation based on simple variables available in EHRs. | High Accuracy and Reliability, Enhanced Efficiency | Limited Model Robustness |
| Jurek J, 2020 (158) | Poland | Convolutional Neural Networks | Clinical | Convolutional Neural Networks, Deep Neural Networks | Propose and evaluate a method using a CNN for reconstructing high-resolution isotropic volumes from 3D MR images with highly anisotropic voxels. | High Accuracy and Reliability, Universal Applicability | Algorithm Complexity and Limitation |
| Li Q, 2022 (159) | China | Pre-trained language models | Research | Deep Learning | Explore methods for integrating imprecise knowledge into prompt-tuning verbalization techniques within the context of biomedical text to stimulate the rich knowledge distributed in PLMs to better serve downstream tasks such as relation extraction. | High Accuracy and Reliability | Algorithm Complexity and Limitation |
| Li X, 2023 (160) | China | Bio-entity2vec and bio-doc2vec | Research | Natural Language Processing | Tracking biomedical articles along the translational continuum from the massive biomedical papers in real-time. | Enhanced Efficiency | Limited Applicability in real-time clinical practice |
| Liu ZT, 2022 (161) | China | Deep Learning | Research | Deep Learning | Enable the exploration of transient biomedical phenomena with high temporal resolution and content-aware image reconstruction. | High Accuracy and Reliability, Enhanced Efficiency | Limited Model Robustness |
| Luo Y, 2015 (162) | USA | Natural Language Processing & Frequent Subgraph Mining | Research | Machine Learning | SANTF used to combat scalability limitations and selection biases, by targeting unsupervised learning for modeling clinical narrative text and mining higher-order features from clinical narrative text, aiming at improving both accuracy and interpretability. | Enhanced Efficiency | Algorithm Complexity and Limitation |
| Luo Y, 2022 (163) | USA | Deep Learning | Research | Deep Learning | Develop a computer-free, all-optical image reconstruction method to see through random diffusers at the speed of light. | Universal Applicability | Algorithm Complexity and Limitation |
| Mesbah S, 2018 (164) | Netherlands | Machine Learning | Research | Machine Learning | Introducing different strategies for training data extraction, semantic expansion, and result entity filtering. | Enhanced Efficiency | Limited Model Robustness |
| Moor M, 2023 (165) | USA | Generalist medical AI | Clinical | Machine Learning | Serves as a foundational model for healthcare, trained on diverse data sets to excel in numerous roles, ranging from disease diagnosis through medical imaging to generating treatment recommendations based on patient data, medical records analysis, and responding to complex medical queries. | Enhanced Efficiency | Algorithm Complexity and Limitation |
| Munshi R, 2006 (166) | USA | Simulation and Visualization | Education | Simulation & Visualization | Introduce and explore the use of simulation and visualization techniques in understanding biological systems at various scales | High Accuracy and Reliability | Limited Applicability in real-time clinical practice |
| Nematzadeh S, 2022 (167) | Türkiye | Machine Learning & Deep Learning | Research | Machine Learning | This study contributes a method to tune hyperparameters of machine learning algorithms using Grey Wolf Optimization (GWO) and Genetic algorithm (GA) metaheuristics, and employ 11 different algorithms on 11 datasets in different biological, biomedical, and nature categories and the results show that in all trials, the performance of the training phases is improved. | Enhanced Efficiency | Not Mentioned in Paper |
| Olimov B, 2021 (168) | South Korea | Convolutional Neural Network | Research | Deep Learning | Introducing a fast and efficient biomedical image segmentation model (FU-NET), emphasizing the use of bottleneck convolution layers for improved performance. | High Accuracy and Reliability | Limited Model Robustness |
| Pang SC, 2019 (169) | China | Convolutional Neural Network | Clinical | Deep Learning | Assist the biomedical image classification task, which combines shallow layer features and deep layer features from the proposed DNN architecture. | Universal Applicability | Algorithm Complexity and Limitation |
| Rivenson Y, 2018 (170) | USA | Convolutional Neural Network | Clinical | Deep Learning | Improve the accuracy and efficiency of mobile-phone microscopy. | Enhanced Efficiency | Limited Applicability in real-time clinical practice |
| Rivera NH, 2023 (171) | USA | Machine Learning | Clinical | Machine Learning | Reduce overuse of ADAMTS13 (AS13) testing for thrombotic thrombocytopenic purpura (TTP), aiding clinical decision-making and resource allocation. | Enhanced Efficiency, High Accuracy and Reliability | Limited Model Robustness |
| Srivastava A, 2022 (172) | Norway | Convolutional Neural Networks | Research | Convolutional Neural Networks | Address challenges in biomedical image segmentation, focusing on efficiently segmenting objects of variable sizes and training on small, biased datasets commonly encountered in biomedical applications. | Enhanced Efficiency, High Accuracy and Reliability, Real-world Clinical Applicability | Algorithm Complexity and Limitation |
| Szijártó A, 2023 (173) | Hungary | Machine Learning | Research | Machine Learning | Introduce a versatile and universal machine learning (ML) framework tailored for the training and evaluation of binary classifiers using tabular biomedical data. | Universal Applicability, Real-world Clinical Applicability | Limited Model Robustness |
| Tang R, 2023 (174) | USA | Deep Learning | Research | Deep Learning, Convolutional Neural Networks | Enhance Image-Activated Cell Sorting (IACS) systems by integrating fast deep learning algorithms for real-time, label-free cell classification and sorting. | Enhanced Efficiency | Algorithm Complexity and Limitation, Limited Model Robustness |
| Tota P, 2021 (175) | Romaina | Natural Language Processing & Telepresence Features | Education | Machine Learning | Proposing a telepresence robot kit containing modules easy to assemble and adapted for various teaching situations, respectively for the development of remote laboratory experiments. | Universal Applicability | Algorithm Complexity and Limitation |
| Weiss R, 2022 (176) | Germany | Deep Learning | Research | Deep Learning | Explore the applications of DL models biomedicine, including image analysis, diagnostics, and biomarker detection. | Real-world Clinical Applicability, Enhanced Efficiency | Algorithm Complexity and Limitation |
| Yan W, 2021 (177) | China | Gradient Boosting Decision Tree, Support Vector Machine, Deep Neural Networks, and Random Forest. | Clinical | Machine Learning & Deep Learning | Develop an AI-driven diagnosis system for early and precise detection of multiple myeloma using routine blood and biochemical tests. | Universal Applicability, High Accuracy and Reliability, Enhanced Efficiency | Algorithm Complexity and Limitation |
| Zhang ZH, 2022 (178) | Japan | Natural Language Processing | Research | Machine Learning | Examine how changes in the ratio of the biomedical domain to general domain data in the corpus affect the extraction of similar biomedical terms using Word2vec. | Enhanced Efficiency | Algorithm Complexity and Limitation |
| Zheng JG, 2015 (179) | USA | Natural Language Processing | Education | Natural Language Processing | Link entities from unstructured full texts of biomedical literature to 300 ontologies. | High Accuracy and Reliability | Algorithm Complexity and Limitation |
| Zheng K, 2015 (180) | USA | Natural Language Processing | Clinical | Natural Language Processing | Assess the ease of adoption of clinical Natural Language Processing (NLP) systems through the development and implementation of usability assessment methods. | Universal Applicability | Moderate Model Accuracy and Reliability, Limited Applicability in real-time clinical practice |
| Zhuang MR, 2022 (181) | China | Annotation-by-iterative-Deep-Learning (AID) | Research | Deep Learning | Streamline the complex process of developing medical image analysis (MIA) algorithms by providing a comprehensive software tool AnatomySketchto assist algorithm developers in training data annotation and GUI construction. | Enhanced Efficiency, Universal Applicability | Limited Model Robustness, Algorithm Complexity and Limitation |
| Yoon JG, 2018 (182) | South Korea | Artificial Neural Networks | Clinical | Machine Learning | Optimize the use of clinical parameters with robustness for DIC diagnosis. | Real-world Clinical Applicability | Limited Model Robustness, Moderate Model Accuracy and Reliability |
| Zhang Z, 2021 (183) | Singapore | Deep Learning | Clinical | Deep learning | Develop fast and noninvasive techniques for characterization and counting of the various cell types used in biomedical applications. | High Accuracy and Reliability | Limited Applicability in real-time clinical practice |
| Henderson N, 2020 (184) | USA | Machine Learning | Education | Machine Learning | Using AI to improve learning environment with enhanced affect responsiveness that will help students in their learning and engagement activities. | High Accuracy and Reliability | Limited Model Robustness |
| Rodriguez-Esteban R, 2022 (185) | Switzerland | Machine Learning | Research | Machine Learning | Predict expert annotations for gating definitions in clinical flow cytometry data. | High Accuracy and Reliability | Limited Model Robustness |
| Segera D, 2020 (186) | Kenya | Ant Colony System (ACS), Improved Discrete Grey Wolf Optimization (IDGWO), and an Excitation mechanism | Research | Machine Learning | Enhance the exploration and exploitation capabilities for or optimal biomedical data feature selection. | Enhanced Efficiency | Algorithm Complexity and Limitation |
| Wang HC, 2021 (187) | China | Machine Learning | Clinical | Machine Learning | Develop and validate an AI autoverification system to support laboratory testing o improve accuracy and efficiency compared to traditional rule-based autoverification models. | High Accuracy and Reliability, Enhanced Efficiency | Algorithm Complexity and Limitation |
| Winfree S, 2022 (188) | USA | Convolutional Neural Networks & Deep Learning | Research | Machine Learning & Deep Learning | Leverage ML and DL techniques to enhance cell segmentation and classification in various imaging modalities to uncover new models of cellular microenvironments in human diseases. | High Accuracy and Reliability, Universal Applicability | Limited Model Robustness |
| Zaman W, 2021 (189) | Malaysia | Machine Learning | Research | Machine Learning & Deep Learning | Apply ML to biomedical research, focusing on stem cell characterization, differentiation, and assessing biosafety and bioefficacy risks in clinical applications. | High Accuracy and Reliability, Enhanced Efficiency | Limited Model Robustness, Algorithm Complexity and Limitation |
| Barrera JAM, 2023 (190) | USA | Machine Learning | Clinical | Machine Learning | Determine PID diagnosis using supervised machine learning algorithms based on classification tree boosting. | Enhanced Efficiency | Limited model robustness |
| Dragomir MA, 2022 (191) | Romania | Machine learning | Clinical | Machine Learning | Develop a model for cell nuclei segmentation in a semi-supervised manner | High Accuracy and Reliability | Algorithm Complexity and Limitation |
| Li H, 2018 (192) | USA | Machine Learning | Research | Machine Learning | Improve effectiveness and accuracy of ML methods by involving users to ultimately improve performance and efficacy of this model. | Enhanced Efficiency | Algorithm Complexity and Limitation |
| Liu HF, 2001 (193) | USA | Machine Learning | Research | Machine Learning | Develop a system that can understand and process complex biomedical terms in written form. | Enhanced Efficiency | Limited Model Robustness |
| Citation | **Country** | **Name**  **of AI** | **Purpose of AI** | **Type**  **of AI** | **Focus of aim** | **Opportunities** | **Limitations** |
| Discipline (8): Serology and immunology | | | | | | | |
| Collatz M, 2021 (194) | Germany | Deep Neural Network | Research | Deep Neural Networks | Streamline and enhance the process of identifying B-cell epitopes on antigenic proteins, crucial for neutralizing pathogens aiming to significantly reduce the time, cost, and labor-intensive nature of epitope detection in comparison to traditional laboratory methods. | High Accuracy and Reliability, Enhanced Efficiency | Limited Applicability in real-time clinical practice, Limited Model Robustness |
| Fey P, 2023 (195) | Germany | Artificial Neural Network | Research | Machine Learning | Explore the feasibility of employing two-dimensional T1/T2 MR relaxometry, with ML as a non-destructive and sterile method for in-process control during the manufacturing and classification process of cell-based treatments. | High Accuracy and Reliability | Limited Model Robustness |
| Giacomini M, 1996 (196) | Italy | Deep Neural Networks | Clinical | Machine Learning | Classify patients with HIV-1 based on their p24 antigen concentration and CD4+ cell counts using neural networks to identify groups with similar profiles for improved clinical follow-up. | Universal Applicability | Algorithm Complexity and Limitation |
| Simonson PD, 2022 (197) | USA | Machine Learning | Clinical | Machine Learning | Automate the process of identifying cases that require additional antibody panels in a clinical flow cytometry lab. | High Accuracy and Reliability, Real-world Clinical Applicability | Not Mentioned in Paper |

Table 2 Categories of scopes* along with the cited articles. AI in biomedical sciences by decades, regions, models, and discipline

| Scopes | Number of articles | Articles | Scope (5): Articles of educational perspective |
| --- | --- | --- | --- |
| Scope (1): AI in biomedical sciences by decades | | | |
| *Pioneering*  *phase*  *(1996-2005)* | 4 | Liu HF 2001, Prank K 2005, Udelhoven T 2000, Giacomini M 1996 |  |
| *Expansion*  *Phase*  *(2006-2015* | 11 | Fernandez-Blanco E 2015, Lippincott T 2011, Luo Y 2015, Munshi R 2006, Zheng JG 2015, Zheng K 2015, Meade A 2009, Karadeniz I 2015, Kotepui M 2015, Richardson AM 2013, Takayama T 2011 | **2** [Munshi R 2006  Zheng JG 2015] |
| *Prosperous phase*  *(2016-2023)* | 177 | Umer 2020, Akbar S 2019, Li RY 2021, Bai BJ 2023, Brázdil T 2022, Flinner N 2022, Ghahremani P 2022, Kundrotas M 2023, Naglah A 2022, Oskal KRJ 2019, Simon O 2018, Smith B 2021, Tellez D 2019, You SX 2019, Zhang HR 2020, Ceran Y 2022, Dhivya P 2022, Li XY 2021, Liang CW 2021, Pellegrino E 2021, Ruini C 2021, Saiz FS 2021, Tellez D 2018, Wu C 2020, Yacob F 2023, Erten M 2023, Farrell CJL 2022, Gerl MJ 2019, Hwang S 2021, Nagai T 2022, Nguyen D 2022, Streun GL 2021, Wilkes EH 2018, Wilkes EH 2020, Yang ZY 2019, Manaka T 2022, Alachram H 2021, Ambite JL 2019, Anthony Q 2021, Aparicio F 2018, Ayad A 2022, Barrera JAM 2023, Barton S 2021, Bonatti AF 2022, Cadamuro J 2023, Chiu PH 2017, Choi H 2023, Demirci F 2016, Dragomir MA 2022, Erdengasileng A 2022, Fillmore N 2019, Goncalves S 2020, He XY 2021, Henderson N 2020, Hill M 2016, Hong LX 2020, Islam MM 2020, Islam MM 2021, Jurek J 2020, Li H 2018, Li Q 2022, Li X 2023, Liu ZT 2022, Luo Y 2022, Mesbah S 2018, Moor M 2023, Nematzadeh S 2022, Olimov B 2021, Pang SC 2019, Rivenson Y 2018, Rivera NH 2023, Rodriguez-Esteban R 2022, Segera D 2020, Srivastava A 2022, Szijártó A 2023, Tang R 2023, Tota P 2021, Wang HC 2021, Weiss R 2022, Winfree S 2022, Yan W 2021, Yoon JG 2018, Zaman W 2021, Zhang Z 2021, Zhang ZH 2022, Zhuang MR 2022, Birjan Z 2023, Buza K 2016, Favalli V 2021, Marceddu G 2019, Price C 2023, Wills JW 2021, Alam MM 2019, Barrera K 2023, Barrera K 2023, Doan M 2020, Eckardt JN 2023, Haung Z 2020, Hou J 2023, Kumari A 2023, Lychagov VV 2023, Nozaka H 2023, Peng KY 2023, Pfeil J 2022, Rodellar J 2022, Rosales MA 2022, Sadafi A 2021, Salama ME 2022, Syed-Abdul S 2020, Uçucu S 2022, Zhou R 2022, Cope S 2022, Flores E 2023, Gao Z 2019, Nesaragi N 2021, Sunil Mohan 2018, Xue WY 2020, Abdulkareem KH 2022, AlJame M 2021, Al-Mualemi BY 2021, Asakura K 2018, Babenko D 2021, Beadell B 2023, Benkwitz-Bedford S 2021, Brinati D 2020, Ceccon DM 2023, Chadaga K 2022, Choi BK 2023, Cihan P 2022, Couret J 2020, Çubukçu H 2021, Feng SY 2022, Ghamdi MAA 2023, Grossi M 2019, Hernandez B 2017, Hou HX 2021, Huang L 2018, Huang SW 2020, Huang TS 2023, Jansen P 2022, Karunakaran V 2022, Kong PH 2022, Kuo PC 2020, Leite DMC 2018, Liu X 2021, Mathison BA 2020, McGuire R 2021, Murugan R 2021, Nakasi R 2021, Normand AC 2022, Pettinati MJ 2020, Ponce P 2022, Priyatharshini R 2021, Quoc VT 2023, Ratzinger F 2018, Rohanian O 2023, Roland T 2022, Savardi M 2018, Shewajo FA 2023, Signoroni A 2018, Signoroni A 2023, Suratanee A 2021, Suri S 2022, Turra G 2017, Tyagin I 2022, Van Den Berg M 2023, Wei ZY 2023, Wu LP 2023, Xu JB 2023, Yasir M 2022, Zeng Y 2023, Zhang F 2023, Zhang YM 2023, Zurac S 2022, Collatz M 2021, Fey P 2023, Simonson PD 2022, Xue WY 2020. | **4** [Ambite JL 2019, Aparicio F 2018, Henderson N 2020, Tota P 2021] |
| Scope (2): AI in biomedical sciences by countries | | | |
| *Low-income* | 4 | Alam MM 2019, Shewajo FA 2023, Abdulkareem KH 2022, Umer 2020 |  |
| *Middle-income* | 54 | Ceccon DM 2023, Li XY 2021, Yang ZY 2019, He XY 2021, Hong LX 2020, Li Q 2022, Li X 2023, Liu ZT 2022, Pang SC 2019, Wang HC 2021, Yan W 2021, Zhuang MR 2022, Hou J 2023, Peng KY 2023, Zhou R 2022, Gao Z 2019, Xue WY 2020, Al-Mualemi BY 2021, Feng SY 2022, Hou HX 2021, Huang L 2018, Liu X 2021, Wei ZY 2023, Zeng Y 2023, Zhang F 2023, Wu LP 2023, Dhivya P 2022, Kumari A 2023, Nesaragi N 2021, Chadaga K 2022, Karunakaran V 2022, Murugan R 2021, Priyatharshini R 2021, Birjan Z 2023, Babenko D 2021, Segera D 2020, Ponce P 2022, Rosales MA 2022, Tota P 2021, Zurac S 2022, Dragomir MA 2022, Lychagov VV 2023, Manaka T 2022, Kotepui M 2015, Suratanee A 2021, Demirci F 2016, Uçucu S 2022, Karadeniz I 2015, Brinati D 2020, Cihan P 2022, Çubukçu H 2021, Nematzadeh S 2022, Nakasi R 2021, Quoc VT 2023, Xue WY 2020 | **1** [Tota P 2021] |
| *High-income* | 134 | Pellegrino E 2021, Szijártó A 2023, Kundrotas M 2023, Farrell CJL 2022, Richardson AM 2013, Erten M 2023, Ratzinger F 2018, Roland T 2022, Akbar S 2019, Pettinati MJ 2020, Brázdil T 2022, Lippincott T 2011, Rohanian O 2023, Gerl MJ 2019, Normand AC 2022, Ruini C 2021, Ayad A 2022, Weiss R 2022, Prank K 2005, Eckardt JN 2023, Sadafi A 2021, Udelhoven T 2000, Collatz M 2021, Fey P 2023, Flinner N 2022, Hill M 2016, Pfeil J 2022, Jansen P 2022, Buza K 2016, Barton S 2021, Meade A 2009, Bonatti AF 2022, Cadamuro J 2023, Favalli V 2021, Grossi M 2019, Savardi M 2018, Signoroni A 2018, Signoroni A 2023, Turra G 2017, Giacomini M 1996, Marceddu G 2019, Nagai T 2022, Zhang ZH 2022, Nozaka H 2023, Asakura K 2018, Takayama T 2011, AlJame M 2021, Zaman W 2021, Tellez D 2019, Tellez D 2018, Mesbah S 2018, Van Den Berg M 2023, Oskal KRJ 2019, Srivastava A 2022, Jurek J 2020, Goncalves S 2020, Hwang S 2021, Ghamdi MAA 2023, Yasir M 2022, Zhang Z 2021, Olimov B 2021, Yoon JG 2018, Syed-Abdul S 2020, Choi BK 2023, Aparicio F 2018, Fernandez-Blanco E 2015, Barrera K 2023, Barrera K 2023, Flores E 2023, Rodellar J 2022, Yacob F 2023, Streun GL 2021, Rodriguez-Esteban R 2022, Leite DMC 2018, Liang CW 2021, Alachram H 2021, Islam MM 2020, Islam MM 2021, Huang SW 2020, Huang TS 2023, Kong PH 2022, Kuo PC 2020, Zhang YM 2023, Zhang HR 2020, Wilkes EH 2018, Wilkes EH 2020, Wills JW 2021, Benkwitz-Bedford S 2021, Hernandez B 2017, Xu JB 2023, Price C 2023, Li RY 2021, Bai BJ 2023, Ghahremani P 2022, Naglah A 2022, Simon O 2018, Smith B 2021, You SX 2019, Ceran Y 2022, Saiz FS 2021, Wu C 2020, Nguyen D 2022, Ambite JL 2019, Anthony Q 2021, Barrera JAM 2023, Chiu PH 2017, Choi H 2023, Erdengasileng A 2022, Fillmore N 2019, Henderson N 2020, Hotz CS 2005, Li H 2018, Liu HF 2001, Luo Y 2015, Luo Y 2022, Moor M 2023, Munshi R 2006, Rivenson Y 2018, Rivera NH 2023, Tang R 2023, Winfree S 2022, Zheng JG 2015, Zheng K 2015, Doan M 2020, Haung Z 2020, Salama ME 2022, Cope S 2022, Sunil Mohan 2018, Beadell B 2023, Couret J 2020, Mathison BA 2020, McGuire R 2021, Suri S 2022, Tyagin I 2022, Simonson PD 2022 | **5** [Aparicio F 2018  Ambite JL 2019  Henderson N 2020  Munshi R 2006  Zheng JG 2015] |
| Scope (3): AI in biomedical sciences by models | | | |
| *Convolutional neural networks* | 21 | Flinner N 2022, Oskal KRJ 2019, Tellez D 2019, Ruini C 2021, Saiz FS 2021, Nagai T 2022, Yang ZY 2019, Barton S 2021, Jurek J 2020, Peng KY 2023, Sadafi A 2021, Couret J 2020, Hou HX 2021, Huang L 2018, Jansen P 2022, Kuo PC 2020, Mathison BA 2020, Normand AC 2022, Pettinati MJ 2020, Signoroni A 2023, Tellez D 2018, Srivastava A 2022, Signoroni A 2018, Tyagin I 2022 |  |
| *Deep learning* | 47 | Akbar S 2019, Bai BJ 2023, Ghahremani P 2022, Naglah A 2022, Smith B 2021, You SX 2019, Zhang HR 2020, Ceran Y 2022, Dhivya P 2022, Yacob F 2023, Farrell CJL 2022, Anthony Q 2021, Ayad A 2022, Goncalves S 2020, He XY 2021, Islam MM 2020, Islam MM 2021, Li Q 2022, Liu ZT 2022, Luo Y 2022, Olimov B 2021, Pang SC 2019, Rivenson Y 2018, Weiss R 2022, Zhang Z 2021, Zhuang MR 2022, Price C 2023, Wills JW 2021, Alam MM 2019, Barrera K 2023, Doan M 2020, Hou J 2023, Nozaka H 2023, Pfeil J 2022, Rodellar J 2022, Sunil Mohan 2018, Xue WY 2020, Abdulkareem KH 2022, Al-Mualemi BY 2021, Feng SY 2022, Nakasi R 2021, Priyatharshini R 2021, Savardi M 2018, Shewajo FA 2023, Takayama T 2011, Turra G 2017, Umer 2020 |  |
| *Machine learning* | 81 | Li RY 2021, Simon O 2018, Pellegrino E 2021, Wu C 2020, Erten M 2023, Gerl MJ 2019, Wilkes EH 2018, Wilkes EH 2020, Ambite JL 2019, Barrera JAM 2023, Chiu PH 2017, Choi H 2023, Dragomir MA 2022, Fillmore N 2019, Henderson N 2020, Hill M 2016, Hong LX 2020, Li H 2018, Liu HF 2001, Luo Y 2015, Mesbah S 2018, Moor M 2023, Nematzadeh S 2022, Rivera NH 2023, Rodriguez-Esteban R 2022, Segera D 2020, Szijártó A 2023, Tota P 2021, Wang HC 2021, Yoon JG 2018, Zhang ZH 2022, Buza K 2016, Favalli V 2021, Marceddu G 2019, Prank K 2005, Eckardt JN 2023, Haung Z 2020, Lychagov VV 2023, Meade A 2009, Rosales MA 2022, Zhou R 2022, Cope S 2022, Flores E 2023, Nesaragi N 2021, AlJame M 2021, Asakura K 2018, Babenko D 2021, Beadell B 2023, Benkwitz-Bedford S 2021, Brinati D 2020, Ceccon DM 2023, Chadaga K 2022, Cihan P 2022, Çubukçu H 2021, Hernandez B 2017, Huang SW 2020, Karunakaran V 2022, Kong PH 2022, Kotepui M 2015, Leite DMC 2018, Liu X 2021, McGuire R 2021, Ponce P 2022, Quoc VT 2023, Ratzinger F 2018, Richardson AM 2013, Rohanian O 2023, Roland T 2022, Suratanee A 2021, Suri S 2022, Van Den Berg M 2023, Wei ZY 2023, Wu LP 2023, Xu JB 2023, Yasir M 2022, Zeng Y 2023, Zhang F 2023, Zhang YM 2023, Fey P 2023, Giacomini M 1996, Simonson PD 2022 | **3** [Ambite JL, 2019  Henderson N, 2020  Tota P, 2021] |
| *Hybrid* | 25 | Brázdil T 2022, Kundrotas M 2023, Liang CW 2021, Saiz FS 2021, Hwang S 2021, Nguyen D 2022, Manaka T 2022, Alachram H 2021, Bonatti AF 2022, Demirci F 2016, Jurek J 2020, Lippincott T 2011, Tang R 2023, Winfree S 2022, Yan W 2021, Zaman W 2021, Birjan Z 2023, Kumari A 2023, Syed-Abdul S 2020, Uçucu S 2022, Gao Z 2019, Choi BK 2023, Ghamdi MAA 2023, Huang TS 2023, Tyagin I 2022 |  |
| *Artificial neural networks* | 2 | Udelhoven T 2000, Fernandez-Blanco E 2015 |  |
| *Automated AI-based diagnosis method* | 2 | Zurac S 2022, Grossi M 2019 |  |
| *Deep neural networks* | 4 | Streun GL 2021, Salama ME 2022, Murugan R 2021, Collatz M 2021 |  |
| *Generative adversarial networks* | 1 | Barrera K, 2023 |  |
| *Knowledge discovery and semantic analysis* | 1 | Li XY, 2021 |  |
| *Natural language processing* | 7 | Zheng K 2015, Aparicio F 2018, Zheng JG 2015, Cadamuro J 2023, Erdengasileng A 2022, Li X 2023, Karadeniz I 2015 | Aparicio F, 2018  Zheng JG, 2015 |
| *Simulation & Visualization* | 1 | Munshi R, 2006 | Munshi R, 2006 |
| *Scope (4): AI in biomedical sciences by discipline.* | | | |
| *Microbiology and infectious diseases* | 63 | Abdulkareem KH 2022, AlJame M 2021, Al-Mualemi BY 2021, Asakura K 2018, Babenko D 2021, Beadell B 2023, Benkwitz-Bedford S 2021, Brinati D 2020, Ceccon DM 2023, Chadaga K 2022, Choi BK 2023, Cihan P 2022, Couret J 2020, Çubukçu H 2021, Feng SY 2022, Ghamdi MAA 2023, Grossi M 2019, Hernandez B 2017, Hou HX 2021, Huang L 2018, Huang SW 2020, Huang TS 2023, Jansen P 2022, Karadeniz I 2015, Karunakaran V 2022, Kong PH 2022, Kotepui M 2015, Kuo PC 2020, Leite DMC 2018, Liu X 2021, Mathison BA 2020, McGuire R 2021, Murugan R 2021, Nakasi R 2021, Normand AC 2022, Pettinati MJ 2020, Ponce P 2022, Priyatharshini R 2021, Quoc VT 2023, Ratzinger F 2018, Richardson AM 2013, Rohanian O 2023, Roland T 2022, Savardi M 2018, Shewajo FA 2023, Signoroni A 2018, Signoroni A 2023, Suratanee A 2021, Suri S 2022, Takayama T 2011, Turra G 2017, Tyagin I 2022, Udelhoven T 2000, Umer 2020, Van Den Berg M 2023, Wei ZY 2023, Wu LP 2023, Xu JB 2023, Yasir M 2022, Zeng Y 2023, Zhang F 2023, Zhang YM 2023, Zurac S 2022 |  |
| *Oncology, Histotechnology, Cytogynecoholgy* | 24 | Akbar S 2019, Li RY 2021, Bai BJ 2023, Brázdil T 2022, Flinner N 2022, Ghahremani P 2022, Kundrotas M 2023, Naglah A 2022, Oskal KRJ 2019, Simon O 2018, Smith B 2021, Tellez D 2019, You SX 2019, Zhang HR 2020, Ceran Y 2022, Dhivya P 2022, Li XY 2021, Liang CW 2021, Pellegrino E 2021, Ruini C 2021, Saiz FS 2021, Tellez D 2018, Wu C 2020, Yacob F 2023 |  |
| *Clinical chemistry* | 10 | Erten M 2023, Farrell CJL 2022, Gerl MJ 2019, Hwang S 2021, Nagai T 2022, Nguyen D 2022, Streun GL 2021, Wilkes EH 2018, Wilkes EH 2020, Yang ZY 2019 |  |
| *Genetics and forensic* | 8 | Manaka T 2022, Birjan Z 2023, Buza K 2016, Favalli V 2021, Marceddu G 2019, Prank K 2005, Price C 2023, Wills JW 2021 |  |
| *Laboratory management* | 6 | Cope S 2022, Flores E 2023, Gao Z 2019, Nesaragi N 2021, Sunil Mohan 2018, Xue WY 2020 |  |
| *Hematology and blood bank* | 20 | , Alam MM 2019, Barrera K 2023, Barrera K 2023, Doan M 2020, Eckardt JN 2023, Haung Z 2020, Hou J 2023, Kumari A 2023, Lychagov VV 2023, Meade A 2009, Nozaka H 2023, Peng KY 2023, Pfeil J 2022, Rodellar J 2022, Rosales MA 2022, Sadafi A 2021, Salama ME 2022, Syed-Abdul S 2020, Uçucu S 2022, Zhou R 2022 |  |
| *General Laboratory* | 57 | Alachram H 2021, Ambite JL 2019, Anthony Q 2021, Aparicio F 2018, Ayad A 2022, Barrera JAM 2023, Barton S 2021, Bonatti AF 2022, Cadamuro J 2023, Chiu PH 2017, Choi H 2023, Demirci F 2016, Dragomir MA 2022, Erdengasileng A 2022, Fernandez-Blanco E 2015, Fillmore N 2019, Goncalves S 2020, He XY 2021, Henderson N 2020, Hill M 2016, Hong LX 2020, Hotz CS 2005, Islam MM 2020, Islam MM 2021, Jurek J 2020, Li H 2018, Li Q 2022, Li X 2023, Lippincott T 2011, Liu HF 2001, Liu ZT 2022, Luo Y 2015, Luo Y 2022, Mesbah S 2018, Moor M 2023, Munshi R 2006, Nematzadeh S 2022, Olimov B 2021, Pang SC 2019, Rivenson Y 2018, Rivera NH 2023, Rodriguez-Esteban R 2022, Segera D 2020, Srivastava A 2022, Szijártó A 2023, Tang R 2023, Tota P 2021, Wang HC 2021, Weiss R 2022, Winfree S 2022, Yan W 2021, Yoon JG 2018, Zaman W 2021, Zhang Z 2021, Zhang ZH 2022, Zheng JG 2015, Zheng K 2015, Zhuang MR 2022 | 6 [Ambite JL 2019, Aparicio F 2018, Henderson N 2020, Munshi R 2006, Tota P 2021, Zheng JG 2015] |
| *Serology and immunology* | 4 | Collatz M 2021, Fey P 2023, Giacomini M 1996, Simonson PD 2022 |  |

** Scope five crosses with all the other scopes. Scope six is not addressed in the table as multiple categories of opportunities and limitations are given for each publication.*

1. Abdulkareem KH, Mostafa SA, Al-Qudsy ZN, Mohammed MA, Al-Waisy AS, Kadry S, et al. Automated System for Identifying COVID-19 Infections in Computed Tomography Images Using Deep Learning Models. Journal of Healthcare Engineering. 2022;2022.

2. Beadell B, Nehra S, Gusenov E, Huse H, Wong-Beringer A. Machine Learning with Alpha Toxin Phenotype to Predict Clinical Outcome in Patients with <i>Staphylococcus aureus</i> Bloodstream Infection. Toxins. 2023;15(7).

3. Huang L, Wu T. Novel neural network application for bacterial colony classification. Theoretical biology & medical modelling. 2018;15(1):22.

4. Normand AC, Chaline A, Mohammad N, Godmer A, Acherar A, Huguenin A, et al. Identification of a clonal population of Aspergillus flavus by MALDI-TOF mass spectrometry using deep learning. Scientific reports. 2022;12(1):1575.

5. AlJame M, Imtiaz A, Ahmad I, Mohammed A. Deep forest model for diagnosing COVID-19 from routine blood tests. Scientific Reports. 2021;11(1).

6. Al-Mualemi BY, Lu L. A Deep Learning-Based Sepsis Estimation Scheme. Ieee Access. 2021;9:5442-52.

7. Asakura K, Azechi T, Sasano H, Matsui H, Hanaki H, Miyazaki M, et al. Rapid and easy detection of low-level resistance to vancomycin in methicillin-resistant <i>Staphylococcus aureus</i> by matrix-assisted laser desorption ionization time-of-flight mass spectrometry. Plos One. 2018;13(3).

8. Babenko D, Seidullayeva A, Bayesheva D, Turdalina B, Omarkulov B, Almabayeva A, et al. Ability of Procalcitonin and C-Reactive Protein for Discriminating between Bacterial and Enteroviral Meningitis in Children Using Decision Tree. Biomed Research International. 2021;2021.

9. Benkwitz-Bedford S, Palm M, Demirtas TY, Mustonen V, Farewell A, Warringer J, et al. Machine Learning Prediction of Resistance to Subinhibitory Antimicrobial Concentrations from <i>Escherichia coli</i> Genomes. Msystems. 2021;6(4).

10. Brinati D, Campagner A, Ferrari D, Locatelli M, Banfi G, Cabitza F. Detection of COVID-19 Infection from Routine Blood Exams with Machine Learning: A Feasibility Study. Journal of Medical Systems. 2020;44(8).

11. Chadaga K, Chakraborty C, Prabhu S, Umakanth S, Bhat V, Sampathila N. Clinical and Laboratory Approach to Diagnose COVID-19 Using Machine Learning. Interdisciplinary Sciences-Computational Life Sciences. 2022;14(2):452-70.

12. Portillo-Lara R, Tahirbegi B, Chapman CAR, Goding JA, Green RA. Mind the gap: State-of-the-art technologies and applications for EEG-based brain-computer interfaces. Apl Bioengineering. 2021;5(3).

13. Cihan P, Ozger ZB. A new approach for determining SARS-CoV-2 epitopes using machine learning-based in silico methods. Computational Biology and Chemistry. 2022;98.

14. Couret J, Moreira DC, Bernier D, Loberti AM, Dotson EM, Alvarez M. Delimiting cryptic morphological variation among human malaria vector species using convolutional neural networks. Plos Neglected Tropical Diseases. 2020;14(12).

15. Çubukçu HC, Topcu DI, Bayraktar N, Gülsen M, Sari N, Arslan AH. Detection of COVID-19 by Machine Learning Using Routine Laboratory Tests. American Journal of Clinical Pathology. 2022;157(5):758-66.

16. Feng SY, Wu ZX, Liang WF, Zhang X, Cai XJ, Li JC, et al. Prediction of Antibiotic Resistance Evolution by Growth Measurement of All Proximal Mutants of Beta-Lactamase. Molecular Biology and Evolution. 2022;39(5).

17. Ghamdi MAA. Identification of Tuberculosis and Coronavirus Patients Using Hybrid Deep Learning Models. Cmc-Computers Materials & Continua. 2023;76(1):881-94.

18. Grossi M, Parolin C, Vitali B, Riccò B. Computer Vision Approach for the Determination of Microbial Concentration and Growth Kinetics Using a Low Cost Sensor System. Sensors. 2019;19(24).

19. Hernandez B, Herrero P, Rawson TM, Moore LSP, Charani E, Holmes AH, et al., editors. Data-drivenWeb-based Intelligent Decision Support System for Infection Management at Point-Of-Care: Case-Based Reasoning Benefits and Limitations. 10th International Joint Conference on Biomedical Engineering Systems and Technologies; 2017 Feb 21-23; Porto, PORTUGAL2017.

20. Hou HX, Cao YK, Cui XX, Liu Z, Xu HJ, Wang C, et al. Medical image management and analysis system based on web for fungal keratitis images. Mathematical Biosciences and Engineering. 2021;18(4):3667-79.

21. Huang SW, Tsai HP, Hung SJ, Ko WC, Wang JR. Assessing the risk of dengue severity using demographic information and laboratory test results with machine learning. PLoS Neglected Tropical Diseases. 2020;14(12):1-19.

22. Huang TS, Wang K, Ye XY, Chen CS, Chang FC. Attention-Guided Transfer Learning for Identification of Filamentous Fungi Encountered in the Clinical Laboratory. Microbiol Spectr. 2023;11(3):e0461122.

23. Jansen P, Creosteanu A, Matyas V, Dilling A, Pina A, Saggini A, et al. Deep Learning Assisted Diagnosis of Onychomycosis on Whole-Slide Images. Journal of Fungi. 2022;8(9).

24. Karadeniz I, Özgür A. Detection and categorization of bacteria habitats using shallow linguistic analysis. Bmc Bioinformatics. 2015;16.

25. Karunakaran V, Joseph MM, Yadev I, Sharma H, Shamna K, Saurav S, et al. A non-invasive ultrasensitive diagnostic approach for COVID-19 infection using salivary label-free SERS fingerprinting and artificial intelligence. Journal of Photochemistry and Photobiology B-Biology. 2022;234.

26. Kong PH, Chiang CH, Lin TC, Kuo SC, Li CF, Hsiung CA, et al. Discrimination of Methicillin-Resistant Staphylococcus aureus by MALDI-TOF Mass Spectrometry with Machine Learning Techniques in Patients with Staphylococcus aureus Bacteremia. Pathogens. 2022;11(5).

27. Kotepui M, Uthaisar K, Phunphuech B, Phiwklam N. A diagnostic tool for malaria based on computer software. Scientific Reports. 2015;5.

28. Kuo PC, Cheng HY, Chen PF, Liu YL, Kang MT, Kuo MC, et al. Assessment of Expert-Level Automated Detection of Plasmodium falciparum in Digitized Thin Blood Smear Images. Jama Network Open. 2020;3(2).

29. Leite DMC, Brochet X, Resch G, Que YA, Neves A, Peña-Reyes C. Computational prediction of inter-species relationships through omics data analysis and machine learning. Bmc Bioinformatics. 2018;19.

30. Mathison BA, Kohan JL, Walker JF, Smith RB, Ardon O, Couturier MR. Detection of Intestinal Protozoa in Trichrome-Stained Stool Specimens by Use of a Deep Convolutional Neural Network. Journal of Clinical Microbiology. 2020;58(6).

31. McGuire RJ, Yu SC, Payne PRO, Lai AM, Vazquez-Guillamet MC, Kollef MH, et al. A Pragmatic Machine Learning Model To Predict Carbapenem Resistance. Antimicrobial Agents and Chemotherapy. 2021;65(7).

32. Murugan R, Goel T, Mirjalili S, Chakrabartty DK. WOANet: Whale optimized deep neural network for the classification of COVID-19 from radiography images. Biocybernetics and Biomedical Engineering. 2021;41(4):1702-18.

33. Nakasi R, Mwebaze E, Zawedde A. Mobile-Aware Deep Learning Algorithms for Malaria Parasites and White Blood Cells Localization in Thick Blood Smears. Algorithms. 2021;14(1).

34. Quoc VT, Ngoc DNT, Hoang TN, Thi HV, Duc MT, Nguyet TD, et al. Predicting Antibiotic Resistance in ICUs Patients by Applying Machine Learning in Vietnam. Infection and Drug Resistance. 2023;16:5535-46.

35. Ratzinger F, Haslacher H, Perkmann T, Pinzan M, Anner P, Makristathis A, et al. Machine learning for fast identification of bacteraemia in SIRS patients treated on standard care wards: a cohort study. Scientific Reports. 2018;8.

36. Rohanian O, Kouchaki S, Soltan A, Yang JY, Rohanian M, Yang Y, et al. Privacy-Aware Early Detection of COVID-19 Through Adversarial Training. Ieee Journal of Biomedical and Health Informatics. 2023;27(3):1249-58.

37. Roland T, Böck C, Tschoellitsch T, Maletzky A, Hochreiter S, Meier J, et al. Domain Shifts in Machine Learning Based Covid-19 Diagnosis From Blood Tests. Journal of Medical Systems. 2022;46(5).

38. Savardi M, Benini S, Signoroni A, editors. β-Hemolysis Detection on Cultured Blood Agar Plates by Convolutional Neural Networks. 21st International Conference on Medical Image Computing and Computer Assisted Intervention (MICCAI); 2018 Sep 16-20; Granada, SPAIN2018.

39. Shewajo FA, Fante KA. Tile-based microscopic image processing for malaria screening using a deep learning approach. Bmc Medical Imaging. 2023;23(1).

40. Signoroni A, Savardi M, Pezzoni M, Guerrini F, Arrigoni S, Turra G. Combining the use of CNN classification and strength-driven compression for the robust identification of bacterial species on hyperspectral culture plate images. Iet Computer Vision. 2018;12(7):941-9.

41. Signoroni A, Ferrari A, Lombardi S, Savardi M, Fontana S, Culbreath K. Hierarchical AI enables global interpretation of culture plates in the era of digital microbiology. Nature Communications. 2023;14(1).

42. Smith KP, Richmond DL, Brennan-Krohn T, Elliott HL, Kirby JE. Development of MAST: A Microscopy-Based Antimicrobial Susceptibility Testing Platform. Slas Technology. 2017;22(6):662-74.

43. Smith KP, Kang AD, Kirby JE. Automated interpretation of blood culture gram stains by use of a deep convolutional neural network. Journal of Clinical Microbiology. 2018;56(3).

44. Suratanee A, Buaboocha T, Plaimas K. Prediction of Human-<i>Plasmodium vivax</i> Protein Associations From Heterogeneous Network Structures Based on Machine-Learning Approach. Bioinformatics and Biology Insights. 2021;15.

45. Suri S, Dakshanamurthy S. IntegralVac: A Machine Learning-Based Comprehensive Multivalent Epitope Vaccine Design Method. Vaccines. 2022;10(10).

46. Turra G, Arrigoni S, Signoroni A, editors. CNN-Based Identification of Hyperspectral Bacterial Signatures for Digital Microbiology. 19th International Conference on Image Analysis and Processing (ICIAP); 2017 Sep 11-15; Catania, ITALY2017.

47. Tyagin I, Kulshrestha A, Sybrandt J, Matta K, Shtutman M, Safro I, et al., editors. Accelerating COVID-19 Research with Graph Mining and Transformer-Based Learning. 36th AAAI Conference on Artificial Intelligence / 34th Conference on Innovative Applications of Artificial Intelligence / 12th Symposium on Educational Advances in Artificial Intelligence; 2022 Feb 22-Mar 01; Electr Network2022.

48. Udelhoven T, Naumann D, Schmitt J. Development of a hierarchical classification system with artificial neural networks and FT-IR spectra for the identification of bacteria. Applied Spectroscopy. 2000;54(10):1471-9.

49. Umer M, Sadiq S, Ahmad M, Ullah S, Choi GS, Mehmood A. A Novel Stacked CNN for Malarial Parasite Detection in Thin Blood Smear Images. Ieee Access. 2020;8:93782-92.

50. Wei TT, Zhang JF, Cheng Z, Jiang L, Li JY, Zhou L. Development and validation of a machine learning model for differential diagnosis of malignant pleural effusion using routine laboratory data. Therapeutic Advances in Respiratory Disease. 2023;17.

51. Wu LP, Xia DD, Xu K. Multi-Clinical Factors Combined with an Artificial Intelligence Algorithm Diagnosis Model for HIV-Infected People with Bloodstream Infection. Infection and Drug Resistance. 2023;16:6085-97.

52. Xu JB, Luo YJ, Wang JK, Tu WM, Yi XF, Xu XG, et al. Artificial intelligence-aided rapid and accurate identification of clinical fungal infections by single-cell Raman spectroscopy. Frontiers in Microbiology. 2023;14.

53. Zeng Y, Wang C, Ye Q, Liu G, Zhang LX, Wan JJ, et al. Machine learning model of imipenem-resistant <i>Klebsiella</i> <i>pneumoniae</i> based on MALDI-TOF-MS platform: An observational study. Health Science Reports. 2023;6(9).

54. Zhang F, Wang H, Liu LY, Su T, Ji B. Machine learning model for the prediction of gram-positive and gram-negative bacterial bloodstream infection based on routine laboratory parameters. Bmc Infectious Diseases. 2023;23(1).

55. Zhang YM, Tsao MF, Chang CY, Lin KT, Keller JJ, Lin HC. Rapid identification of carbapenem-resistant Klebsiella pneumoniae based on matrix-assisted laser desorption ionization time-of-flight mass spectrometry and an artificial neural network model. Journal of Biomedical Science. 2023;30(1).

56. Ceccon DM, Amaral PHR, Andrade LM, da Silva MIN, Andrade LAF, Moraes TFS, et al. New, fast, and precise method of COVID-19 detection in nasopharyngeal and tracheal aspirate samples combining optical spectroscopy and machine learning. Brazilian Journal of Microbiology. 2023;54(2):769-77.

57. Liu X, Su TJF, Hsu YMS, Yu H, Yang HS, Jiang L, et al. Rapid identification and discrimination of methicillin-resistant <i>Staphylococcus aureus</i> strains via matrix-assisted laser desorption/ionization time-of-flight mass spectrometry. Rapid Communications in Mass Spectrometry. 2021;35(2).

58. Priyatharshini R, Aswath RAS, Sreenidhi MN, Joshi SS, Dhandapani R, Ieee, editors. An Efficient Approach for Automatic detection of COVID-19 using Transfer Learning from Chest X-Ray Images. 3rd International Conference on Signal Processing and Communication (ICPSC); 2021 May 13-14; Coimbatore, INDIA2021.

59. Yasir M, Karim AM, Malik SK, Bajaffer AA, Azhar EI. Application of Decision-Tree-Based Machine Learning Algorithms for Prediction of Antimicrobial Resistance. Antibiotics. 2022;11(11).

60. Pettinati MJ, Chen GB, Rajput KS, Selvaraj N, Ieee, editors. Practical Machine Learning-Based Sepsis Prediction. 42nd Annual International Conference of the IEEE-Engineering-in-Medicine-and-Biology-Society (EMBC); 2020 Jul 20-24; Montreal, CANADA2020.

61. Richardson AM, Lidbury BA. Infection status outcome, machine learning method and virus type interact to affect the optimised prediction of hepatitis virus immunoassay results from routine pathology laboratory assays in unbalanced data. Bmc Bioinformatics. 2013;14.

62. Takayama T, Ebinuma H, Tada S, Yamagishi Y, Wakabayashi K, Ojiro K, et al. Prediction of Effect of Pegylated Interferon Alpha-2b plus Ribavirin Combination Therapy in Patients with Chronic Hepatitis C Infection. Plos One. 2011;6(12).

63. van den Berg M, Medina O, Loohuis I, van der Flier M, Dudink J, Benders M, et al. Development and clinical impact assessment of a machine-learning model for early prediction of late-onset sepsis. Computers in Biology and Medicine. 2023;163.

64. Ponce P, Mata O, Perez E, Lopez JR, Molina A, McDaniel T. S4 Features and Artificial Intelligence for Designing a Robot against COVID-19-Robocov. Future Internet. 2022;14(1).

65. Nesaragi N, Patidar S, Thangaraj V. A correlation matrix-based tensor decomposition method for early prediction of sepsis from clinical data. Biocybernetics and Biomedical Engineering. 2021;41(3):1013-24.

66. Chiu PH, Hripcsak G. EHR-based phenotyping: Bulk learning and evaluation. Journal of Biomedical Informatics. 2017;70:35-51.

67. Zurac S, Mogodici C, Poncu T, Trascau M, Popp C, Nichita L, et al. A New Artificial Intelligence-Based Method for Identifying Mycobacterium Tuberculosis in Ziehl-Neelsen Stain on Tissue. Diagnostics. 2022;12(6).

68. Smith B, Hermsen M, Lesser E, Ravichandar D, Kremers W. Developing image analysis pipelines of whole-slide images: Pre- and post-processing. Journal of Clinical and Translational Science. 2021;5(1).

69. Akbar S, Peikari M, Salama S, Panah AY, Nofech-Mozes S, Martel AL. Automated and Manual Quantification of Tumour Cellularity in Digital Slides for Tumour Burden Assessment. Scientific Reports. 2019;9.

70. Bai BJ, Yang XL, Li YZ, Zhang YJ, Pillar N, Ozcan A. Deep learning-enabled virtual histological staining of biological samples. Light-Science & Applications. 2023;12(1).

71. Brázdil T, Gallo M, Nenutil R, Kubanda A, Toufar M, Holub P. Automated annotations of epithelial cells and stroma in hematoxylin-eosin-stained whole-slide images using cytokeratin re-staining. Journal of Pathology Clinical Research. 2022;8(2):129-42.

72. Ceran Y, Ergüder H, Ladner K, Korenfeld S, Deniz K, Padmanabhan S, et al. TNTdetect.AI: A Deep Learning Model for Automated Detection and Counting of Tunneling Nanotubes in Microscopy Images. Cancers. 2022;14(19).

73. Flinner N, Gretser S, Quaas A, Bankov K, Stoll A, Heckmann LE, et al. Deep learning based on hematoxylin-eosin staining outperforms immunohistochemistry in predicting molecular subtypes of gastric adenocarcinoma. Journal of Pathology. 2022;257(2):218-26.

74. Ghahremani P, Li YY, Kaufman A, Vanguri R, Greenwald N, Angelo M, et al. Deep learning-inferred multiplex immunofluorescence for immunohistochemical image quantification. Nature Machine Intelligence. 2022;4(4):401-+.

75. Kundrotas M, Mazoniene E, Sesok D. Automatic Tumor Identification from Scans of Histopathological Tissues. Applied Sciences-Basel. 2023;13(7).

76. Li RY, Gujja S, Bajaj SR, Gamel OE, Cilfone N, Gulcher JR, et al. Quantum processor-inspired machine learning in the biomedical sciences. Patterns. 2021;2(6).

77. Li XY, Peng SY, Du J. Towards medical knowmetrics: representing and computing medical knowledge using semantic predications as the knowledge unit and the uncertainty as the knowledge context. Scientometrics. 2021;126(7):6225-51.

78. Liang CW, Fang PW, Huang HY, Lo CM. Deep Convolutional Neural Networks Detect Tumor Genotype from Pathological Tissue Images in Gastrointestinal Stromal Tumors. Cancers. 2021;13(22).

79. Naglah A, Khalifa F, El-Baz A, Gondim D. Conditional GANs based system for fibrosis detection and quantification in Hematoxylin and Eosin whole slide images. Medical Image Analysis. 2022;81.

80. Oskal KRJ, Risdal M, Janssen EAM, Undersrud ES, Gulsrud TO. A U-net based approach to epidermal tissue segmentation in whole slide histopathological images. Sn Applied Sciences. 2019;1(7).

81. Pellegrino E, Jacques C, Beaufils N, Nanni I, Carlioz A, Metellus P, et al. Machine learning random forest for predicting oncosomatic variant NGS analysis. Scientific Reports. 2021;11(1).

82. Ruini C, Schlingmann S, Jonke Z, Avci P, Padrón-Laso V, Neumeier F, et al. Machine Learning Based Prediction of Squamous Cell Carcinoma in Ex Vivo Confocal Laser Scanning Microscopy. Cancers. 2021;13(21).

83. Saiz FS, Sanders C, Stevens R, Nielsen R, Britt M, Yuravlivker L, et al. Artificial Intelligence Clinical Evidence Engine for Automatic Identification Prioritization, and Extraction of Relevant Clinical Oncology Research. Jco Clinical Cancer Informatics. 2021;5:102-11.

84. Simon O, Yacoub R, Jain S, Tomaszewski JE, Sarder P. Multi-radial LBP Features as a Tool for Rapid Glomerular Detection and Assessment in Whole Slide Histopathology Images. Scientific Reports. 2018;8.

85. Tellez D, Balkenhol M, Otte-Höller I, van de Loo R, Vogels R, Bult P, et al. Whole-Slide Mitosis Detection in H&E Breast Histology Using PHH3 as a Reference to Train Distilled Stain-Invariant Convolutional Networks. Ieee Transactions on Medical Imaging. 2018;37(9):2126-36.

86. Tellez D, Litjens G, Bándi P, Bulten W, Bokhorst JM, Ciompi F, et al. Quantifying the effects of data augmentation and stain color normalization in convolutional neural networks for computational pathology. Medical Image Analysis. 2019;58.

87. Wu C, Zhao XN, Welsh M, Costello K, Cao KJ, Abou Tayoun A, et al. Using Machine Learning to Identify True Somatic Variants from Next-Generation Sequencing. Clinical Chemistry. 2020;66(1):239-46.

88. Zhang HR, Kalirai H, Acha-Sagredo A, Yang XY, Zheng YL, Coupland SE. Piloting a Deep Learning Model for Predicting Nuclear BAP1 Immunohistochemical Expression of Uveal Melanoma from Hematoxylin-and-Eosin Sections. Translational Vision Science & Technology. 2020;9(2).

89. Dhivya P, Kumaresan T, Subramanian P, Gunasekaran K, Kumar GS. HYBRID FIREFLY META OPTIMIZATION FOR BIO MEDICAL IMAGE PROCESSING USING DEEP LEARNING. Journal of Pharmaceutical Negative Results. 2022;13(4):1199-209.

90. Yacob F, Siarov J, Villiamsson K, Suvilehto JT, Sjöblom L, Kjellberg M, et al. Weakly supervised detection and classification of basal cell carcinoma using graph-transformer on whole slide images. Scientific Reports. 2023;13(1).

91. You SX, Sun Y, Yang L, Park J, Tu HH, Marjanovic M, et al. Real-time intraoperative diagnosis by deep neural network driven multiphoton virtual histology. Npj Precision Oncology. 2019;3.

92. Farrell CJL. Decision support or autonomous artificial intelligence? The case of wrong blood in tube errors. Clinical Chemistry and Laboratory Medicine. 2022;60(12):1993-7.

93. Gerl MJ, Klose C, Surma MA, Fernandez C, Melander O, Männistö S, et al. Machine learning of human plasma lipidomes for obesity estimation in a large population cohort. PLoS Biology. 2019;17(10).

94. Hwang S, Gwon C, Seo DM, Cho J, Kim JY, Uh Y. A Deep Neural Network for Estimating Low-Density Lipoprotein Cholesterol From Electronic Health Records: Real-Time Routine Clinical Application. Jmir Medical Informatics. 2021;9(8).

95. Nagai T, Onodera O, Okuda S. Deep learning classification of urinary sediment crystals with optimal parameter tuning. Scientific Reports. 2022;12(1).

96. Streun GL, Steuer AE, Ebert LC, Dobay A, Kraemer T. Interpretable machine learning model to detect chemically adulterated urine samples analyzed by high resolution mass spectrometry. Clinical Chemistry and Laboratory Medicine. 2021;59(8):1392-9.

97. Yang ZY, Leng L, Kim BG. StoolNet for Color Classification of Stool Medical Images. Electronics. 2019;8(12).

98. Wilkes EH, Emmett E, Beltran L, Woodward GM, Carling RS. A Machine Learning Approach for the Automated Interpretation of Plasma Amino Acid Profiles. Clinical Chemistry. 2020;66(9):1210-8.

99. Wilkes EH, Rumsby G, Woodward GM. Using machine learning to aid the interpretation of urine steroid profiles. Clinical Chemistry. 2018;64(11):1586-95.

100. Nguyen D, Tao L, Li Y. Integration of Machine Learning and Coarse-Grained Molecular Simulations for Polymer Materials: Physical Understandings and Molecular Design. Frontiers in Chemistry. 2022;9.

101. Erten M, Barua PD, Tuncer I, Dogan S, Baygin M, Tuncer T, et al. Swin-LBP: a competitive feature engineering model for urine sediment classification. Neural Computing & Applications. 2023;35(29):21621-32.

102. Birjan Z, Khashei Varnamkhasti K, Parhoudeh S, Naeimi L, Naeimi S. Crucial Role of Foxp3 Gene Expression and Mutation in Systemic Lupus Erythematosus, Inferred from Computational and Experimental Approaches. Diagnostics. 2023;13(22).

103. Buza K. Classification of gene expression data: A hubness-aware semi-supervised approach. Computer Methods and Programs in Biomedicine. 2016;127:105-13.

104. Favalli V, Tini G, Bonetti E, Vozza G, Guida A, Gandini S, et al. Machine learning-based reclassification of germline variants of unknown significance: The RENOVO algorithm. American Journal of Human Genetics. 2021;108(4):682-95.

105. Manaka T, van Zyl T, Kar D, editors. Improving Cause-of-Death Classification from Verbal Autopsy Reports. Third Southern African Conference, SACAIR; 2022 Dec 05-09; Stellenbosch, SOUTH AFRICA2022.

106. Marceddu G, Dallavilla T, Guerri G, Zulian A, Marinelli C, Bertelli M. Analysis of machine learning algorithms as integrative tools for validation of next generation sequencing data. European Review for Medical and Pharmacological Sciences. 2019;23(18):8139-47.

107. Prank K, Schulze E, Eckert O, Nattkemper TW, Bettendorf M, Maser-Gluth C, et al. Machine learning approaches for phenotype-genotype mapping:: predicting heterozygous mutations in the CYP21B gene from steroid profiles. European Journal of Endocrinology. 2005;153(2):301-5.

108. Price C, Russell JA. AMAnD: an automated metagenome anomaly detection methodology utilizing DeepSVDD neural networks. Frontiers in Public Health. 2023;11.

109. Wills JW, Verma JR, Rees BJ, Harte DSG, Haxhiraj Q, Barnes CM, et al. Inter-laboratory automation of the in vitro micronucleus assay using imaging flow cytometry and deep learning. Archives of Toxicology. 2021;95(9):3101-15.

110. Flores E, Blasco Á, Carbonell R, López-Garrigós M, Torreblanca R, Martinez-Racaj L, et al. ANOTHER STEP FORWARD CLINICAL LABORATORY A DECISION MAKER HUB: PREDICTING URINARY TRACT INFECTIONS IN THE EMERGENCY DEPARTMENT WITH MACHINE LEARNING. Clinical Chemistry and Laboratory Medicine. 2023;61:S339.

111. Gao Z, Fu G, Ouyang CP, Tsutsui S, Liu XZ, Yang J, et al. edge2vec: Representation learning using edge semantics for biomedical knowledge discovery. Bmc Bioinformatics. 2019;20.

112. Gordon MM, Moser AM, Rubin E. Unsupervised Analysis of Classical Biomedical Markers: Robustness and Medical Relevance of Patient Clustering Using Bioinformatics Tools. Plos One. 2012;7(3).

113. Mohan S, Fiorini N, Kim S, Lu ZY, Assoc Comp M, editors. A Fast Deep Learning Model for Textual Relevance in Biomedical Information Retrieval. 27th World Wide Web (WWW) Conference; 2018 Apr 23-27; Lyon, FRANCE2018.

114. Xue WY, Li QY, Xue QY. Text Detection and Recognition for Images of Medical Laboratory Reports With a Deep Learning Approach. Ieee Access. 2020;8:407-16.

115. Cope S. AI infrastructure for the digitization and automation of clinical laboratories. Clinica Chimica Acta. 2022;530:S243.

116. Alam MM, Islam MT. Machine learning approach of automatic identification and counting of blood cells. Healthcare Technology Letters. 2019;6(4):103-8.

117. Barrera K, Merino A, Molina A, Rodellar J. Automatic generation of artificial images of leukocytes and leukemic cells using generative adversarial networks (syntheticcellgan). Computer Methods and Programs in Biomedicine. 2023;229.

118. Barrera K, Rodellar J, Alferez S, Merino A. Automatic normalized digital color staining in the recognition of abnormal blood cells using generative adversarial networks. Computer Methods and Programs in Biomedicine. 2023;240.

119. Eckardt JN, Röllig C, Metzeler K, Heisig P, Stasik S, Georgi JA, et al. Unsupervised meta-clustering identifies risk clusters in acute myeloid leukemia based on clinical and genetic profiles. Communications Medicine. 2023;3(1).

120. Hou J, Ren W, Zhao W, Li H, Liu M, Wang H, et al. Blood clot and fibrin recognition method for serum images based on deep learning. Clin Chim Acta. 2023;553:117732.

121. Kumari A, Kumari A, Singh A, Singh SK, Juhi A, Dhanvijay AKD, et al. Large Language Models in Hematology Case Solving: A Comparative Study of ChatGPT-3.5, Google Bard, and Microsoft Bing. Cureus Journal of Medical Science. 2023;15(8).

122. Lychagov VV, Semenov VM, Volkova EK, Chernakov DI, Ahn J, Kim JY. Noninvasive Hemoglobin Measurements With Photoplethysmography in Wrist. Ieee Access. 2023;11:79636-47.

123. Meade AD, Clarke C, Bonnier F, Poon K, Garcia A, Knief P, et al., editors. FUNCTIONAL AND PATHOLOGICAL ANALYSIS OF BIOLOGICAL SYSTEMS USING VIBRATIONAL SPECTROSCOPY WITH CHEMOMETRIC AND HEURISTIC APPROACHES. 1st Workshop on Hyperspectral Image and Signal Processing - Evolution in Remote Sensing; 2009 Aug 26-29; Grenoble, FRANCE2009.

124. Nozaka H, Kushibiki M, Kamata K, Yamagata K. Classifying Microscopic Images of Reactive Lymphocytosis Using Two-Step Tandem AI Models. Applied Sciences-Basel. 2023;13(9).

125. Peng KY, Peng YH, Liao HD, Yang ZS, Feng WL. Automated bone marrow cell classification through dual attention gates dense neural networks. Journal of Cancer Research and Clinical Oncology. 2023.

126. Pfeil J, Nechyporenko A, Frohme M, Hufert FT, Schulze K. Examination of blood samples using deep learning and mobile microscopy. BMC bioinformatics. 2022;23(1):65.

127. Rodellar J, Barrera K, Alférez S, Boldú L, Laguna J, Molina A, et al. A Deep Learning Approach for the Morphological Recognition of Reactive Lymphocytes in Patients with COVID-19 Infection. Bioengineering-Basel. 2022;9(5).

128. Rosales MA, de Luna RG. Computer-Based Blood Type Identification Using Image Processing and Machine Learning Algorithm. Journal of Advanced Computational Intelligence and Intelligent Informatics. 2022;26(5):698-705.

129. Sadafi A, Makhro A, Livshits L, Navab N, Bogdanova A, Albarqouni S, et al., editors. Sickle Cell Disease Severity Prediction from Percoll Gradient Images Using Graph Convolutional Networks. 3rd MICCAI Workshop on Domain Adaptation and Representation Transfer (DART); 2021 Sep 27-Oct 01; Strasbourg, FRANCE2021.

130. Salama ME, Otteson GE, Camp JJ, Seheult JN, Jevremovic D, Holmes DR, et al. Artificial Intelligence Enhances Diagnostic Flow Cytometry Workflow in the Detection of Minimal Residual Disease of Chronic Lymphocytic Leukemia. Cancers. 2022;14(10).

131. Syed-Abdul S, Firdani RP, Chung HJ, Uddin M, Hur M, Park JH, et al. Artificial Intelligence based Models for Screening of Hematologic Malignancies using Cell Population Data. Scientific reports. 2020;10(1):4583.

132. Uçucu S, Karablylk T, Azik FM. Machine learning models can predict the presence of variants in hemoglobin: Artificial neural network-based recognition of human hemoglobin variants by HPLC. Turkish Journal of Biochemistry. 2022.

133. Zhou R, Liang YF, Cheng HL, Wang W, Huang DW, Wang Z, et al. A highly accurate delta check method using deep learning for detection of sample mix-up in the clinical laboratory. Clinical Chemistry and Laboratory Medicine. 2022;60(12):1984-92.

134. Bransky A, Larsson A, Aardal E, Ben-Yosef Y, Christenson RH. A Novel Approach to Hematology Testing at the Point of Care. Journal of Applied Laboratory Medicine. 2021;6(2):532-42.

135. Doan M, Sebastian JA, Caicedo JC, Siegert S, Roch A, Turner TR, et al. Objective assessment of stored blood quality by deep learning. Proceedings of the National Academy of Sciences of the United States of America. 2020;117(35):21381-90.

136. Huang Z, Siddhanta S, Zheng G, Kickler T, Barman I. Rapid, Label-free Optical Spectroscopy Platform for Diagnosis of Heparin-Induced Thrombocytopenia. Angewandte Chemie (International ed in English). 2020;59(15):5972-8.

137. Islam MM, Poly TN, Yang HC, Li YC. Deep into Laboratory: An Artificial Intelligence Approach to Recommend Laboratory Tests. Diagnostics. 2021;11(6).

138. Lippincott T, Séaghdha DO, Korhonen A. Exploring subdomain variation in biomedical language. Bmc Bioinformatics. 2011;12.

139. Alachram H, Chereda H, Beissbarth T, Wingender E, Stegmaier P. Text mining-based word representations for biomedical data analysis and protein-protein interaction networks in machine learning tasks. Plos One. 2021;16(10).

140. Ambite JL, Gordon J, Fierro L, Burns G, Mathew J, Aaai, editors. Linking Educational Resources on Data Science. 33rd AAAI Conference on Artificial Intelligence / 31st Innovative Applications of Artificial Intelligence Conference / 9th AAAI Symposium on Educational Advances in Artificial Intelligence; 2019 Jan 27-Feb 01; Honolulu, HI2019.

141. Anthony Q, Xu L, Subramoni H, Panda DKD, Ieee, editors. Scaling Single-Image Super-Resolution Training on Modern HPC Clusters: Early Experiences. 35th IEEE International Parallel and Distributed Processing Symposium (IPDPS); 2021 Jun 17-21; Portland, OR2021.

142. Aparicio F, Morales-Botello ML, Rubio M, Hernando A, Muñoz R, López-Fernández H, et al. Perceptions of the use of intelligent information access systems in university level active learning activities among teachers of biomedical subjects. International Journal of Medical Informatics. 2018;112:21-33.

143. Ayad A, Hallawa A, Peine A, Martin L, Fazlic LB, Dartmann G, et al. Predicting Abnormalities in Laboratory Values of Patients in the Intensive Care Unit Using Different Deep Learning Models: Comparative Study. Jmir Medical Informatics. 2022;10(8).

144. Barton S, Alakkari S, O'Dwyer K, Ward T, Hennelly B. Convolution Network with Custom Loss Function for the Denoising of Low SNR Raman Spectra. Sensors. 2021;21(14).

145. Bonatti AF, Vozzi G, Chua CK, De Maria C. A Deep Learning Quality Control Loop of the Extrusion-based Bioprinting Process. International Journal of Bioprinting. 2022;8(4):307-20.

146. Cadamuro J, Cabitza F, Debeljak Z, De Bruyne S, Frans G, Perez SM, et al. Potentials and pitfalls of ChatGPT and natural-language artificial intelligence models for the understanding of laboratory medicine test results. An assessment by the European Federation of Clinical Chemistry and Laboratory Medicine (EFLM) Working Group on Artificial Intelligence (WG-AI). Clinical Chemistry and Laboratory Medicine. 2023;61(7):1158-66.

147. Choi H, Moran J, Matsumoto N, Hernandez ME, Moore JH. Aliro: an automated machine learning tool leveraging large language models. Bioinformatics. 2023;39(10).

148. Demirci F, Akan P, Kume T, Sisman AR, Erbayraktar Z, Sevinc S. Artificial Neural Network Approach in Laboratory Test Reporting:  Learning Algorithms. Am J Clin Pathol. 2016;146(2):227-37.

149. Erdengasileng A, Han Q, Zhao T, Tian S, Sui X, Li K, et al. Pre-trained models, data augmentation, and ensemble learning for biomedical information extraction and document classification. Database : the journal of biological databases and curation. 2022;2022.

150. Fernandez-Blanco E, Rivero D, Gestal M, Fernández-Lozano C, Ezquerra N, Munteanu CR, et al. A Hybrid Evolutionary System for Automated Artificial Neural Networks Generation and Simplification in Biomedical Applications. Current Bioinformatics. 2015;10(5):672-91.

151. Fillmore N, Do N, Brophy M, Zimolzak A. Interactive Machine Learning for Laboratory Data Integration. Stud Health Technol Inform. 2019;264:133-7.

152. Goncalves S, Cortez P, Moro S. A deep learning classifier for sentence classification in biomedical and computer science abstracts. Neural Computing & Applications. 2020;32(11):6793-807.

153. He XY, Yu B, Ren YG. SWACG: A Hybrid Neural Network Integrating Sliding Window for Biomedical Event Trigger Extraction. Journal of Imaging Science and Technology. 2021;65(6).

154. Hill M, Hoena B, Kilian W, Odenwald S, editors. Wearable, modular and intelligent sensor laboratory. 11th Conference of the International Sports Engineering Association (ISEA); 2016 Jul 11-14; Delft, NETHERLANDS2016.

155. Hong LX, Lin JJ, Li SY, Wan FP, Yang H, Jiang T, et al. A novel machine learning framework for automated biomedical relation extraction from large-scale literature repositories. Nature Machine Intelligence. 2020;2(6):347-+.

156. Hotz CS, Templeton SJ, Christopher MM. Comparative analysis of expert and machine-learning methods for classification of body cavity effusions in companion animals. Journal of Veterinary Diagnostic Investigation. 2005;17(2):158-64.

157. Islam MM, Yang HC, Poly TN, Li YCJ. Development of an Artificial Intelligence-Based Automated Recommendation System for Clinical Laboratory Tests: Retrospective Analysis of the National Health Insurance Database. Jmir Medical Informatics. 2020;8(11).

158. Jurek J, Kocinski M, Materka A, Elgalal M, Majos A. CNN-based superresolution reconstruction of 3D MR images using thick-slice scans. Biocybernetics and Biomedical Engineering. 2020;40(1):111-25.

159. Li Q, Wang YC, You T, Lu YT. BioKnowPrompt: Incorporating imprecise knowledge into prompt-tuning verbalizer with biomedical text for relation extraction. Information Sciences. 2022;617:346-58.

160. Li X, Tang XL, Lu W. Tracking biomedical articles along the translational continuum: a measure based on biomedical knowledge representation. Scientometrics. 2023;128(2):1295-319.

161. Liu ZT, Wang LL, Meng Y, He TT, He SF, Yang YS, et al. All-fiber high-speed image detection enabled by deep learning. Nature Communications. 2022;13(1).

162. Luo Y, Xin Y, Hochberg E, Joshi R, Uzuner O, Szolovits P. Subgraph augmented non-negative tensor factorization (SANTF) for modeling clinical narrative text. Journal of the American Medical Informatics Association. 2015;22(5):1009-19.

163. Luo Y, Zhao YF, Li JX, Cetintas E, Rivenson Y, Jarrahi M, et al. Computational imaging without a computer: seeing through random diffusers at the speed of light. Elight. 2022;2(1).

164. Mesbah S, Lofi C, Torre MV, Bozzon A, Houben GJ, editors. TSE-NER: An Iterative Approach for Long-Tail Entity Extraction in Scientific Publications. 17th International Semantic Web Conference (ISWC); 2018 Oct 08-12; Monterey, CA2018.

165. Moor M, Banerjee O, Abad ZSH, Krumholz HM, Leskovec J, Topol EJ, et al. Foundation models for generalist medical artificial intelligence. Nature. 2023;616(7956):259-65.

166. Munshi R, Coalson RD, Ermentrout GB, Madura JD, Meirovitch H, Stiles JR, et al. An introduction to simulation and visualization of biological systems at multiple scales: A summer training program for interdisciplinary research. Biotechnology Progress. 2006;22(1):179-85.

167. Nematzadeh S, Kiani F, Torkamanian-Afshar M, Aydin N. Tuning hyperparameters of machine learning algorithms and deep neural networks using metaheuristics: A bioinformatics study on biomedical and biological cases. Computational Biology and Chemistry. 2022;97.

168. Olimov B, Sanjar K, Din S, Ahmad A, Paul A, Kim J. FU-Net: fast biomedical image segmentation model based on bottleneck convolution layers. Multimedia Systems. 2021;27(4):637-50.

169. Pang SC, Du AA, Orgun MA, Yu ZZ. A novel fused convolutional neural network for biomedical image classification. Medical & Biological Engineering & Computing. 2019;57(1):107-21.

170. Rivenson Y, Koydemir HC, Wang HD, Wei ZS, Ren ZS, Günaydin H, et al. Deep Learning Enhanced Mobile-Phone Microscopy. Acs Photonics. 2018;5(6):2354-64.

171. Rivera NH, McClintock DS, Alterman MA, Alterman TAL, Pruitt HD, Olsen GM, et al. A clinical laboratorian's journey in developing a machine learning algorithm to assist in testing utilization and stewardship. Journal of Laboratory and Precision Medicine. 2023;8.

172. Srivastava A, Jha D, Chanda S, Pal U, Johansen H, Johansen D, et al. MSRF-Net: A Multi-Scale Residual Fusion Network for Biomedical Image Segmentation. Ieee Journal of Biomedical and Health Informatics. 2022;26(5):2252-63.

173. Szijártó A, Fábián A, Lakatos BK, Tolvaj M, Merkely B, Kovács A, et al. A machine learning framework for performing binary classification on tabular biomedical data An ML framework for binary classification. IMAGING. 2023;15(1).

174. Tang Q, Shi X, Xu Y, Zhou R, Zhang S, Wang X, et al. Identification and Validation of the Diagnostic Markers for Inflammatory Bowel Disease by Bioinformatics Analysis and Machine Learning. Biochemical Genetics. 2023.

175. Tota P, Vaida MF, Ieee, editors. Modular Telepresence Robot for Distance Medical Education. 9th IEEE International Conference on e-Health and Bioengineering (EHB); 2021 Nov 18-19; Grigore T Popa Univ Med & Pharmacy, ELECTR NETWORK2021.

176. Weiss R, Karimijafarbigloo S, Roggenbuck D, Rödiger S. Applications of Neural Networks in Biomedical Data Analysis. Biomedicines. 2022;10(7).

177. Yan W, Shi H, He T, Chen J, Wang C, Liao AJ, et al. Employment of Artificial Intelligence Based on Routine Laboratory Results for the Early Diagnosis of Multiple Myeloma. Frontiers in Oncology. 2021;11.

178. Zhang ZH, Han F, Zhang HJ, Aoki T, Ogasawara K. Examining the Effect of the Ratio of Biomedical Domain to General Domain Data in Corpus in Biomedical Literature Mining. Applied Sciences-Basel. 2022;12(1).

179. Zheng JG, Howsmon D, Zhang BL, Hahn J, McGuinness D, Hendler J, et al. Entity linking for biomedical literature. Bmc Medical Informatics and Decision Making. 2015;15.

180. Zheng K, Vydiswaran VGV, Liu Y, Wang Y, Stubbs A, Uzuner Ö, et al. Ease of adoption of clinical natural language processing software: An evaluation of five systems. Journal of Biomedical Informatics. 2015;58:S189-S96.

181. Zhuang MR, Chen ZH, Wang HK, Tang H, He J, Qin BB, et al. AnatomySketch: An Extensible Open-Source Software Platform for Medical Image Analysis Algorithm Development. Journal of Digital Imaging. 2022;35(6):1623-33.

182. Yoon JG, Heo J, Kim M, Park YJ, Choi MH, Song J, et al. Machine learning-based diagnosis for disseminated intravascular coagulation (DIC): Development, external validation, and comparison to scoring systems. Plos One. 2018;13(5).

183. Zhang Z, Leong KW, van Vliet K, Barbastathis G, Ravasio A. Deep learning for label-free nuclei detection from implicit phase information of mesenchymal stem cells. Biomedical Optics Express. 2021;12(3):1683-706.

184. Henderson N, Rowe J, Paquette L, Baker RS, Lester J, editors. Improving Affect Detection in Game-Based Learning with Multimodal Data Fusion. 21st International Conference on Artificial Intelligence in Education (AIED); 2020 Jul 06-10; Electr Network2020.

185. Rodriguez-Esteban R, Duarte J, Teixeira PC, Richard F, Koltsova S, So WV. Prediction of standard cell types and functional markers from textual descriptions of flow cytometry gating definitions using machine learning. Cytometry Part B-Clinical Cytometry. 2022;102(3):220-7.

186. Segera D, Mbuthia M, Nyete A. An Innovative Excited-ACS-IDGWO Algorithm for Optimal Biomedical Data Feature Selection. Biomed Research International. 2020;2020.

187. Wang HC, Wang HY, Zhang J, Li XL, Sun CX, Zhang Y. Using machine learning to develop an autoverification system in a clinical biochemistry laboratory. Clinical Chemistry and Laboratory Medicine. 2021;59(5):883-91.

188. Winfree S. User-Accessible Machine Learning Approaches for Cell Segmentation and Analysis in Tissue. Frontiers in Physiology. 2022;13.

189. Zaman W, Karman SB, Ramlan EI, Tukimin SNB, Ahmad MYB. Machine Learning in Stem Cells Research: Application for Biosafety and Bioefficacy Assessment. Ieee Access. 2021;9:25926-45.

190. Barrera JAM, Guzmán SR, Cascajares EH, Garabedian EK, Fuleihan RL, Sullivan KE, et al. Who's your data? Primary immune deficiency differential diagnosis prediction via machine learning and data mining of the USIDNET registry. Clinical Immunology. 2023;255.

191. Dragomir MA, Macinic V, Cojocar GS, editors. A Semi-Supervised Approach to Cell Nuclei Segmentation. IEEE 18th International Conference on Intelligent Computer Communication and Processing (ICCP); 2022 Sep 22-24; Electr Network2022.

192. Li H, Fang SF, Mukhopadhyay S, Saykin AJ, Shen L, editors. Interactive Machine Learning by Visualization: A Small Data Solution. IEEE International Conference on Big Data (Big Data); 2018 Dec 10-13; Seattle, WA2018.

193. Liu HF, Lussier YA, Friedman C. Disambiguating ambiguous biomedical terms in biomedical narrative text: An unsupervised method. Journal of Biomedical Informatics. 2001;34(4):249-61.

194. Collatz M, Mock F, Barth E, Hoelzer M, Sachse K, Marz M. EpiDope: a deep neural network for linear B-cell epitope prediction. Bioinformatics. 2021;37(4):448-55.

195. Fey P, Weber DL, Stebani J, Mörchel P, Jakob P, Hansmann J, et al. Non-destructive classification of unlabeled cells: Combining an automated benchtop magnetic resonance scanner and artificial intelligence. Plos Computational Biology. 2023;19(2).

196. Giacomini M, Ruggiero C, Maillard M, Lillo FB, Varnier OE. Objective evaluation of two markers of HIV-1 infection (p24 antigen concentration and CD4+ cell counts) by a self organizing neural network. Medical Informatics. 1996;21(3):215-28.

197. Simonson PD, Lee AY, Wu D. Potential for Process Improvement of Clinical Flow Cytometry by Incorporating Real-Time Automated Screening of Data to Expedite Addition of Antibody Panels A Single Laboratory Analysis. American Journal of Clinical Pathology. 2022;157(3):443-50.
