# Supplementary material for: Artificial Intelligence in Biomedical Sciences: A Scoping Review
Source: Br J Biomed Sci. 2025 Aug 5;82:14362. doi: 10.3389/bjbs.2025.14362 (PMC12360964; doi:10.3389/bjbs.2025.14362)
Supplement: Supplementary file 2 [file DataSheet1.docx]

# Appendix 1: Search strategies

Embase

| ('artificial intelligence':ab,ti OR 'machine learning':ab,ti OR 'computational intelligence':ab,ti OR 'machine intelligence':ab,ti OR 'computer reasoning':ab,ti OR 'computer vision system*':ab,ti OR 'knowledge acquisition':ab,ti OR 'knowledge representation*':ab,ti OR 'expert system*':ab,ti OR 'intelligent retrieval':ab,ti OR 'knowledge engineering':ab,ti OR 'neural network':ab,ti OR 'natural language processing':ab,ti OR 'deep learning':ab,ti) AND ('clinical laborator*':ab,ti OR 'medical laborator*':ab,ti OR 'biomedical science*':ab,ti OR 'medical laboratory technology':ab,ti OR 'clinical laboratory science*':ab,ti OR 'laboratory patholog*':ab,ti OR 'medical laboratory science*') AND (educat* OR train* OR literac*) |
| --- |

PubMed

((((((((((((((("artificial intelligence"[Title/Abstract]) OR ("machine learning"[Title/Abstract])) OR ("computational intelligence"[Title/Abstract])) OR ("machine intelligence"[Title/Abstract])) OR ("computer reasoning"[Title/Abstract])) OR ("computer vision system*"[Title/Abstract])) OR ("knowledge acquisition"[Title/Abstract])) OR ("knowledge representation*"[Title/Abstract])) OR ("expert system*"[Title/Abstract])) OR ("intelligent retrieval"[Title/Abstract])) OR ("knowledge engineering"[Title/Abstract])) OR ("neural network"[Title/Abstract])) OR ("natural language processing"[Title/Abstract])) OR ("deep learning"[Title/Abstract])) AND ((((((("clinical laborator*"[Title/Abstract]) OR ("medical laborator*"[Title/Abstract])) OR ("biomedical science*"[Title/Abstract])) OR ("medical laboratory technology"[Title/Abstract])) OR ("clinical laboratory science*"[Title/Abstract])) OR ("laboratory patholog*"[Title/Abstract])) OR ("medical laboratory science*"[Title/Abstract]))) AND ((((educat*) OR (education [MeSH Terms])) OR ((train*) OR (training [MeSH Terms]))) OR ((literac*) OR (literacy [MeSH Terms])))

Web of Science

| (TI=('artificial intelligence')) OR AB=('artificial intelligence') | 116996 |
| --- | --- |
| (TI=('machine learning')) OR AB=( 'machine learning') | 343945 |
| (TI=('computational intelligence')) OR AB=( 'computational intelligence') | 14171 |
| (TI=('machine intelligence')) OR AB=( 'machine intelligence') | 32508 |
| (TI=('computer reasoning')) OR AB=( 'computer reasoning') | 17718 |
| (TI=('computer vision system*')) OR AB=( 'computer vision system*') | 23970 |
| (TI=('knowledge acquisition')) OR AB=( 'knowledge acquisition') | 34465 |
| (TI=('knowledge representation*')) OR AB=( 'knowledge representation*') | 57215 |
| (TI=('expert system*')) OR AB=( 'expert system*') | 132399 |
| (TI=('intelligent retrieval')) OR AB=( 'intelligent retrieval') | 2718 |
| (TI=('knowledge engineering')) OR AB=( 'knowledge engineering') | 60262 |
| (TI=('neural network')) OR AB=( 'neural network') | 579554 |
| (TI=('natural language processing')) OR AB=( 'natural language processing') | 36745 |
| (TI=('deep learning')) OR AB=( 'deep learning') | 254595 |
| #1 OR #2 OR #3 OR #4 OR #5 OR #6 OR #7 OR #8 OR #9 OR #10 OR #11 OR #12 OR #13 OR #14 | 1346766 |
| (TI=('clinical laborator*')) OR AB=( 'clinical laborator*') | 194566 |
| (TI=('medical laborator*')) OR AB=( 'medical laborator*') | 50497 |
| (TI=('biomedical science*')) OR AB=( 'biomedical science*') | 17384 |
| (TI=('medical laboratory technology')) OR AB=( 'medical laboratory technology') | 3052 |
| (TI=('clinical laboratory science*')) OR AB=( 'clinical laboratory science*') | 5360 |
| (TI=('laboratory patholog*')) OR AB=( 'laboratory patholog*') | 24892 |
| (TI=('medical laboratory science*')) OR AB=( 'medical laboratory science*') | 3057 |
| #16 OR #17 OR #18 OR #19 OR #20 OR #21 OR #22 | 248529 |
| ((ALL=(educat*)) OR ALL=(train*)) OR ALL=(literac*) | 6312225 |
| #15 AND #23 AND #24 | 2021 |

# Appendix 2: PRESS Guideline


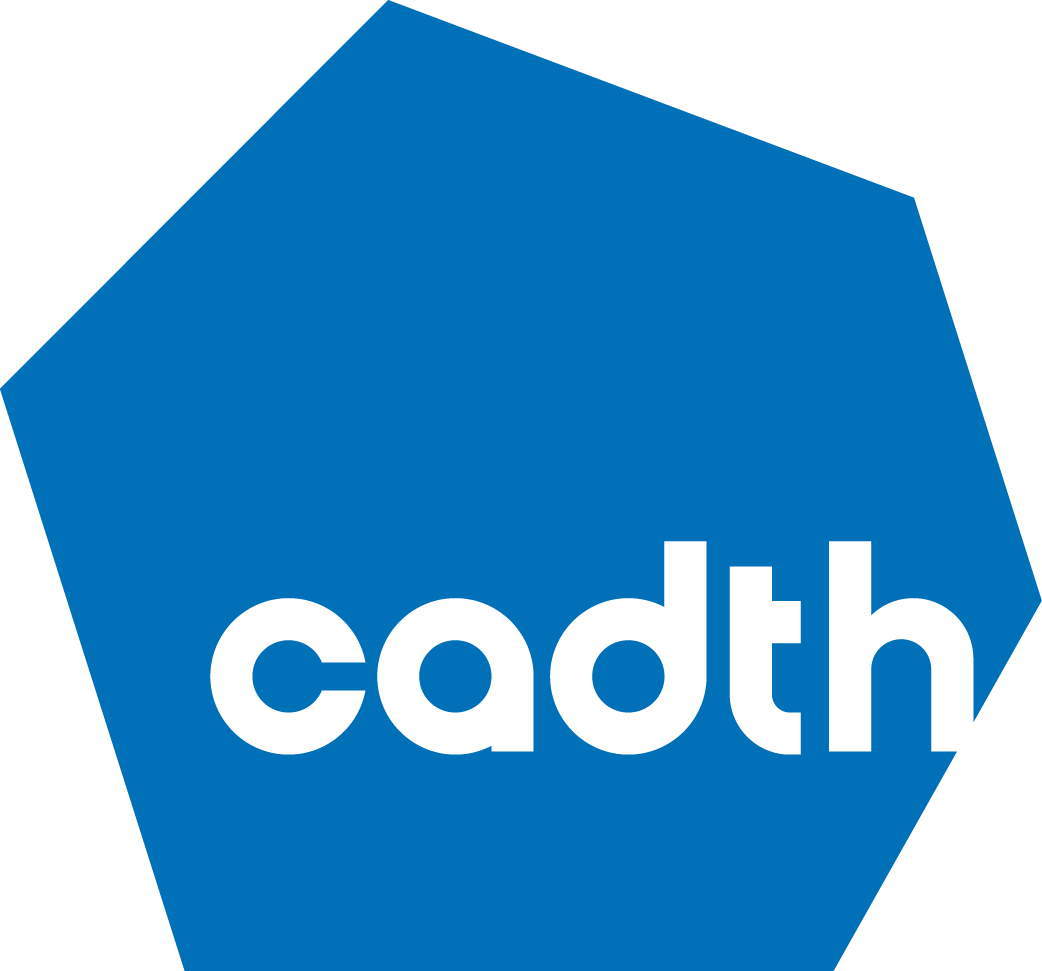


# Table 10: PRESS Guideline — Search Submission and Peer Review Assessment

**Search Submission: This section is to be filled in by the searcher**

**Searcher:** Maha Abdulla H I Al-Asmakh **Email:** maha.alasmakh@qu.edu.qa

**Date submitted: NA Date requested by: NA**

**Search Topic or Title:**

**Artificial Intelligence in Biomedical Sciences: A scoping review**

**This search strategy is:**

My PRIMARY (core) database strategy:

This is my first submission  This is submitted after feedback
 This is an update (the search has been previously used in an evidence syntheses)

**This search strategy is:**

My SECONDARY (supplemental) database strategy:

This is my first submission  This is submitted after feedback
 This is an update (the search has been previously used in an evidence syntheses)

**Database(s)**

(e.g., MEDLINE, CINAHL, Embase): **[mandatory]**

PubMed, Embase, Web of Science

**Database Platform(s)**

(e.g., Ovid, EBSCO): **[mandatory]**

Click or tap here to enter text.

*If your chosen database or platform provides a link to the search history, please provide it here:

Yes, adressed above

**Research Question(s)**

(Describe the purpose of the search) **[mandatory]**

The review answers two questions: what are the characteristics of AI in biomedical sciences? What are the applications of AI in biomedical sciences education?

**PICO(S) or Related Format**

(Outline the PICOs, SPIDER, PEPSI, etc. for your question — i.e., **P**atient, **I**ntervention, **C**omparison, **O**utcome, and **S**tudy Design — as applicable)

**P NA**

**I AI in biomedical sciences**

**C NA**

**O NA**

**S Scoping review**

**Inclusion Criteria**

(List criteria such as age groups, study designs, and so on to be included) [optional]

We included any publication of original research on the application of AI in the biomedical sciences.

**Exclusion Criteria**

(List criteria such as study designs, date limits, and so on to be excluded) [optional]

We excluded any publication not relying on primary data, case reports, case series, editorials, expert opinion, commentaries, reviews, conference abstracts of peer-reviewed publications, and publications not relevant to educational, clinical, and research perspectives of biomedical sciences

**Were Search Filters Applied? [mandatory]**

Yes  No

If YES, which were used (e.g., Cochrane RCT filter, CADTH’s Guidelines filter, PubMed Clinical Queries filter)? Provide the source if this is a published filter. **[mandatory if the answer was YES]**

Click or tap here to enter text.

Other notes or comments you feel would be useful for the peer reviewer (e.g., decision on date or language limits, articles used in pulling search terms)? **[optional]**

Click or tap here to enter text.

Copy and paste your search strategy here, exactly as run, including the number of hits per line. **[mandatory]**

Mentioned above

# Appendix 3: PRSMA- ScR Chesklist

**Preferred Reporting Items for Systematic reviews and Meta-Analyses extension for Scoping Reviews (PRISMA-ScR) Checklist**

| **SECTION** | **ITEM** | **PRISMA-ScR CHECKLIST ITEM** | **REPORTED ON PAGE #** |
| --- | --- | --- | --- |
| **TITLE** | | | |
| Title | 1 | Identify the report as a scoping review. | 1 |
| **ABSTRACT** | | | |
| Structured summary | 2 | Provide a structured summary that includes (as applicable): background, objectives, eligibility criteria, sources of evidence, charting methods, results, and conclusions that relate to the review questions and objectives. | 1,2 |
| **INTRODUCTION** | | | |
| Rationale | 3 | Describe the rationale for the review in the context of what is already known. Explain why the review questions/objectives lend themselves to a scoping review approach. | 3 |
| Objectives | 4 | Provide an explicit statement of the questions and objectives being addressed with reference to their key elements (e.g., population or participants, concepts, and context) or other relevant key elements used to conceptualize the review questions and/or objectives. | 3,4 |
| **METHODS** | | | |
| Protocol and registration | 5 | Indicate whether a review protocol exists; state if and where it can be accessed (e.g., a Web address); and if available, provide registration information, including the registration number. | 4 |
| Eligibility criteria | 6 | Specify characteristics of the sources of evidence used as eligibility criteria (e.g., years considered, language, and publication status), and provide a rationale. | 4 |
| Information sources* | 7 | Describe all information sources in the search (e.g., databases with dates of coverage and contact with authors to identify additional sources), as well as the date the most recent search was executed. | 3,4 |
| Search | 8 | Present the full electronic search strategy for at least 1 database, including any limits used, such that it could be repeated. | 3,4, Appendix 1 |
| Selection of sources of evidence† | 9 | State the process for selecting sources of evidence (i.e., screening and eligibility) included in the scoping review. | 3,4, Appendix 1 |
| Data charting process‡ | 10 | Describe the methods of charting data from the included sources of evidence (e.g., calibrated forms or forms that have been tested by the team before their use, and whether data charting was done independently or in duplicate) and any processes for obtaining and confirming data from investigators. | 3,4 |
| Data items | 11 | List and define all variables for which data were sought and any assumptions and simplifications made. | 3,4 |
| Critical appraisal of individual sources of evidence§ | 12 | If done, provide a rationale for conducting a critical appraisal of included sources of evidence; describe the methods used and how this information was used in any data synthesis (if appropriate). | NA |
| Synthesis of results | 13 | Describe the methods of handling and summarizing the data that were charted. | 4 |
| **RESULTS** | | | |
| Selection of sources of evidence | 14 | Give numbers of sources of evidence screened, assessed for eligibility, and included in the review, with reasons for exclusions at each stage, ideally using a flow diagram. | 5 |
| Characteristics of sources of evidence | 15 | For each source of evidence, present characteristics for which data were charted and provide the citations. | 5 |
| Critical appraisal within sources of evidence | 16 | If done, present data on critical appraisal of included sources of evidence (see item 12). | NA |
| Results of individual sources of evidence | 17 | For each included source of evidence, present the relevant data that were charted that relate to the review questions and objectives. | 5 |
| Synthesis of results | 18 | Summarize and/or present the charting results as they relate to the review questions and objectives. | 5-9 |
| **DISCUSSION** | | | |
| Summary of evidence | 19 | Summarize the main results (including an overview of concepts, themes, and types of evidence available), link to the review questions and objectives, and consider the relevance to key groups. | 9-11 |
| Limitations | 20 | Discuss the limitations of the scoping review process. | 10-11 |
| Conclusions | 21 | Provide a general interpretation of the results with respect to the review questions and objectives, as well as potential implications and/or next steps. | 12 |
| **FUNDING** | | | |
| Funding | 22 | Describe sources of funding for the included sources of evidence, as well as sources of funding for the scoping review. Describe the role of the funders of the scoping review. | 12 |

JBI = Joanna Briggs Institute; PRISMA-ScR = Preferred Reporting Items for Systematic reviews and Meta-Analyses extension for Scoping Reviews.

* Where *sources of evidence* (see second footnote) are compiled from, such as bibliographic databases, social media platforms, and Web sites.

† A more inclusive/heterogeneous term used to account for the different types of evidence or data sources (e.g., quantitative and/or qualitative research, expert opinion, and policy documents) that may be eligible in a scoping review as opposed to only studies. This is not to be confused with *information sources* (see first footnote).

‡ The frameworks by Arksey and O’Malley (6) and Levac and colleagues (7) and the JBI guidance (4, 5) refer to the process of data extraction in a scoping review as data charting*.*

§ The process of systematically examining research evidence to assess its validity, results, and relevance before using it to inform a decision. This term is used for items 12 and 19 instead of "risk of bias" (which is more applicable to systematic reviews of interventions) to include and acknowledge the various sources of evidence that may be used in a scoping review (e.g., quantitative and/or qualitative research, expert opinion, and policy document).

*From:* Tricco AC, Lillie E, Zarin W, O'Brien KK, Colquhoun H, Levac D, et al. PRISMA Extension for Scoping Reviews (PRISMAScR): Checklist and Explanation. Ann Intern Med. 2018;169:467–473. [doi: 10.7326/M18-0850](http://annals.org/aim/fullarticle/2700389/prisma-extension-scoping-reviews-prisma-scr-checklist-explanation).

# Appendix 5: Countries classification by income

|  |  |  |  |
| --- | --- | --- | --- |
|  | low income | middle income | high income |
|  | Bangladesh | China | Australia |
|  | Pakistan | Malaysia | Singapore |
|  | Nepal | Vietnam | Hong Kong |
|  | Ethiopia | Thailand | New Zealand |
|  | Iraq | Indonesia | England |
|  | Iran | Philippines | Canada |
|  |  | Argentina | Sweden |
|  |  | Brazil | Saudi Arabia |
|  |  | Chile | United Arab Emirates |
|  |  | Malaysia | United States of America |
|  |  | Poland | Switzerland |
|  |  | South Africa | Italy |
|  |  | Turkey | Spain |
|  |  | India | Germany |
|  |  | Brazil | Ireland |
|  |  | Romania | South Korea |
|  |  | Russia | Japan |
|  |  | Mexico | Taiwan |
|  |  | Kazakhstan | Netherland |
|  |  |  | Czech Republic |
|  |  |  | Poland |
|  |  |  | Austria |
|  |  |  | Hunary |
|  |  |  | Ireland |
|  |  |  | Lithuania |
|  |  |  | Kuwait |
|  |  |  | Norway |
|  |  |  | Israel |
|  |  |  |  |

McGettigan P, Henry D. Use of non-steroidal anti-inflammatory drugs that elevate cardiovascular risk: an examination of sales and essential medicines lists in low-, middle-, and high-income countries. PLoS medicine. 2013 Feb 12;10(2):e1001388.

Pathadka S, Yan VK, Neoh CF, Al-Badriyeh D, Kong DC, Slavin MA, Cowling BJ, Hung IF, Wong IC, Chan EW. Global consumption trend of antifungal agents in humans from 2008 to 2018: data from 65 middle-and high-income countries. Drugs. 2022 Jul;82(11):1193-205.

# Appendix 6: Opportunities and limitations codes and themes

|  |  |
| --- | --- |
| **Opportunities** | **Categories Under opportunities** |
| High Accuracy and Reliability | Consider Comprehensive Risk Factors |
|  | Internal and external validation success |
|  | Improved Early Detection |
|  | Outperformance of State-of-the-Art Models |
|  | Enhanced Detection Capability |
|  | Evaluator Diversity |
| Real-world Clinical Applicability | High Diagnostic Performance |
|  | Consistent Reliable Results |
|  | Diagnostic Support |
|  | Clinical validation |
| Universal Applicability | Robustness to Variations |
|  | Generalizability across different patient populations |
|  | Comprehensive Parameter Panel |
|  | Domain Shift Identification |
|  | Open-Source Nature |
|  | Model Diversity |
|  | Improved Variant Classification |
|  | Real-World Data |
|  | User-Friendly |
|  | Location and Knowledge Independence |
| Enhanced Efficiency | Time-Efficient in analysis process |
|  | High Predictive Accuracy |
|  | Resource and Cost Reduction |
|  | Automation of Classification |
|  | Parameter Optimization |
|  | Machine Learning Application |
|  | Reduction of Uninterpreted Variants |
| Enhanced Privacy Protection | Reduced Legal and Reputational Risks |
|  |  |
|  |  |
|  |  |
| **Limitations** | **Categories Under Limitations** |
| Moderate Model Accuracy and Reliability | Moderate Precision and Recall |
|  | Algorithm Refinement Needed |
|  | Need for Continuous Adaptation |
| Limited Applicability in real-time clinical practice | Retrospective Nature |
|  | Diagnostic Criteria Variation |
|  | Biological Variability |
|  | Lack of Clinical validation |
| Limited Model Robustness | Single-Center Data |
|  | Limited patient demographics and disease prevalence |
|  | Limited Sample Size |
|  | Limited Model Generalizability |
|  | Dataset Dependency |
|  | Continuous Evolution of COVID-19 |
|  | Dependence on Image Quality |
|  | Dependency on Diagnostic Criteria |
|  | Epitope Diversity |
|  | Dependency on Parameters |
|  | Dependency on User Expertise |
| Algorithm Complexity and Limitation | Methodological Considerations |
|  | Limited Impact of Machine Learning Methods |
|  | Assumption of Binary Classification |
|  | Modest Mortality Prediction |
|  |  |
